# Dysregulation of mitochondria-lysosome contacts by *GBA1* dysfunction in dopaminergic neuronal models of Parkinson’s disease

**DOI:** 10.1038/s41467-021-22113-3

**Published:** 2021-03-22

**Authors:** Soojin Kim, Yvette C. Wong, Fanding Gao, Dimitri Krainc

**Affiliations:** Department of Neurology, Northwestern University Feinberg School of Medicine, Chicago, IL USA

**Keywords:** Lysosomes, Mitochondria, Parkinson's disease

## Abstract

Mitochondria-lysosome contacts are recently identified sites for mediating crosstalk between both organelles, but their role in normal and diseased human neurons remains unknown. In this study, we demonstrate that mitochondria-lysosome contacts can dynamically form in the soma, axons, and dendrites of human neurons, allowing for their bidirectional crosstalk. Parkinson’s disease patient derived neurons harboring mutant *GBA1* exhibited prolonged mitochondria-lysosome contacts due to defective modulation of the untethering protein TBC1D15, which mediates Rab7 GTP hydrolysis for contact untethering. This dysregulation was due to decreased *GBA1* (β-glucocerebrosidase (GCase)) lysosomal enzyme activity in patient derived neurons, and could be rescued by increasing enzyme activity with a GCase modulator. These defects resulted in disrupted mitochondrial distribution and function, and could be further rescued by TBC1D15 in Parkinson’s patient derived *GBA1*-linked neurons. Together, our work demonstrates a potential role of mitochondria-lysosome contacts as an upstream regulator of mitochondrial function and dynamics in midbrain dopaminergic neurons in *GBA1*-linked Parkinson’s disease.

## Abstract (web summary)

Mitochondria-lysosome contact sites mediate cross-talk between the two organelles. Here, the authors show mitochondria-lysosome contacts are prolonged and defective in heterozygous mutant *GBA1* neurons, which is caused by defective modulation of TBC1D15 due to decreased *GBA1* activity.

## Introduction

Mitochondria–lysosome (M–L) contact sites were recently identified as inter-organelle membrane contacts involving the dynamic tethering of mitochondria with lysosomes^1,2^. Importantly, M–L contacts allow for the bidirectional regulation of both mitochondrial and lysosomal network dynamics, and mediate their direct interaction in a pathway distinct from mitophagy or lysosomal degradation of mitochondrial-derived vesicles^2^. M–L contact sites are further regulated by TBC1D15, a Rab7 GAP recruited to mitochondria via the outer mitochondrial membrane protein Fis1^3–6^, which mediates Rab7 GTP hydrolysis to promote M–L contact untethering dynamics^2^. However, the formation and role of M–L contacts in human neurons have not been previously investigated.

Parkinson’s disease (PD) is the most common neurodegenerative movement disorder characterized by progressive loss of dopaminergic neurons in the substantia nigra pars compacta (SNc), leading to the cardinal motor symptoms of PD^7,8^. Interestingly, both mitochondrial and lysosomal defects have been genetically and functionally linked to PD^9,10^. Indeed, the lysosomal enzyme *GBA1* which encodes for β-glucocerebrosidase (GCase) and catalyzes the hydrolysis of glucosylceramide (GlcCer) to glucose and ceramide, represents the greatest genetic risk factor for PD, with *GBA1* mutation carriers exhibiting more severe cognitive symptoms^11,12^. *GBA1-*PD patient-derived mutant neurons demonstrate both lower GCase protein levels and reduced GCase activity^13–16^. Moreover, wild-type GCase activity is decreased in both idiopathic and multiple types of familial PD patient neurons^9,16–20^, highlighting targeting of *GBA1* as a potential therapeutic approach for PD^14,16,21^ and a key role for GCase activity in PD pathogenesis. However, whether M–L contact dynamics are disrupted in PD patient neurons and further modulated by GCase activity is not known.

Here we investigated the impact of M–L contact sites in neuronal function and *GBA1*-linked PD pathogenesis. Using super-resolution time-lapse imaging, we found that M–L contact sites dynamically formed in the soma, axon, and dendrites of human iPSC-derived dopaminergic neurons. We further reported a role for M–L contacts in PD patient-derived dopaminergic neurons, as contact untethering was disrupted in patient neurons expressing heterozygous mutant *GBA1*. Finally, we showed that defective M–L contact untethering was caused by decreased GCase lysosomal enzymatic activity leading to GlcCer accumulation. This resulted in decreased levels of the untethering protein TBC1D15 which normally mediates Rab7 GTP hydrolysis for contact untethering. Ultimately, these defects resulted in disrupted mitochondrial distribution and function, but could be rescued by TBC1D15 in Parkinson’s patient *GBA1*-linked neurons. Together, our work identifies an important role for M–L contact sites in human neurons and in *GBA1*-linked PD pathophysiology.

## Results

### M–L contact sites dynamically form in human neurons

To investigate M–L contact sites in human neurons, we utilized induced pluripotent stem cell (iPSC) technology (Supplementary Fig. 1a) to generate human midbrain dopaminergic neurons from healthy controls using previously established protocols^16,22^. Differentiated neurons expressed the neural specific marker β-III-tubulin (TUJ1) and midbrain dopaminergic neuron-specific markers: tyrosine hydroxylase (TH), forkhead box protein A2 (FOXA2), and LIM Homeobox Transcription Factor 1 Alpha (LMX1A) (Supplementary Fig. 2a, b), and were subsequently imaged for mitochondrial and lysosomal dynamics in live neurons (Supplementary Fig. 3a, b).

**Fig. 1:**
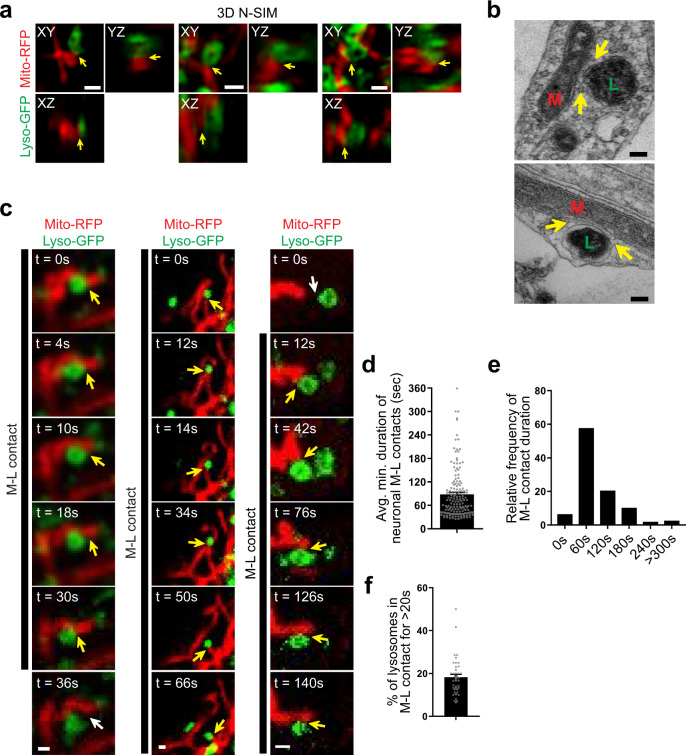
Mitochondria–lysosome contacts dynamically form in human neurons. **a** Representative 3D structured illumination microscopy (N-SIM) images of M–L contacts (yellow arrows) in wild-type human dopaminergic iPSC-derived neurons (mitochondria: red, Mito–RFP; lysosomes: green, Lyso-GFP) (30 neurons from *N* = 3 independent experiments were imaged). **b** Representative electron microscopy (EM) images of M–L contacts (yellow arrows) with distance between membranes <10 nm (mitochondria, M; lysosomes, L). **c** Representative time-lapse confocal images of dynamic contacts between mitochondria (red, Mito-RFP) and lysosomes (green, Lyso-GFP). Time-lapse recordings were taken at 2 s intervals for 3–5 min. Yellow arrows mark stable M–L contacts. White arrows mark the site of M–L contacts before contact formation or after contact untethering. Black line shows duration of contacts (see Supplementary Movies 1 and 2). **d**, **e** Quantification of duration of stable M–L contacts from confocal images (*n* = 156 contacts from 26 neurons, *N* = 3 independent experiments). **d** Average minimum duration of neuronal M–L contacts. **e** Relative frequency (percentages) distribution of neuronal M–L contacts. *X*-axis represents bin centers. Bin width = 60 s. **f** Percentage of lysosomes contacting mitochondria (for >20 s) (*n* = 41 neurons, *N* = 3 independent experiments). For all quantifications, data are means ± S.E.M. Scale bars, 500 nm (**a**, **c**); 100 nm (**b**).

Using 3D super-resolution structured illumination microscopy (3D N-SIM), we found that mitochondria and lysosomes (Mito-RFP, Lyso-GFP) formed stable inter-organelle contacts in human neurons (Fig. 1a). Neuronal M–L contacts were further confirmed to be <10 nm apart using electron microscopy (EM) (Fig. 1b), consistent with other contact sites^2,23^. Next, we conducted confocal time-lapse microscopy of live neurons, and found that M–L contacts dynamically formed over time and remained tethered together (yellow arrows) before subsequently untethering from one another (white arrows) (Fig. 1c and Supplemental Movies 1–3). Mitochondria in contact with lysosomes maintained their membrane potential, as visualized by TMRM imaging (Supplementary Fig. 3c–e), and dynamic M–L contact tethering could be further observed in human neurons by imaging outer mitochondrial membrane and lysosomal membrane proteins (TOM20-RFP, LAMP1-GFP) (Supplementary Fig. 3f), as well as by imaging mitochondrial and lysosomal-targeted dyes (MitoTracker, LysoTracker) (Supplementary Fig. 3g). In addition, we could also visualize M–L contacts in human neurons by proximity ligation assay (PLA) imaging of TOM20 and LAMP1 on the outer mitochondrial and lysosomal membrane (Supplementary Fig. 4a, b). M–L contacts in human neurons remained tethered for an average duration of 88.07 ± 5.05 s (Fig. 1d, e), with ~18% of lysosomes in contact with mitochondria at any point in time (Fig. 1f). Thus, M–L contacts can dynamically form to mediate crosstalk between mitochondria and lysosomes in human neurons.

### Spatial compartmentalization of neuronal M–L contact dynamics

**Fig. 2:**
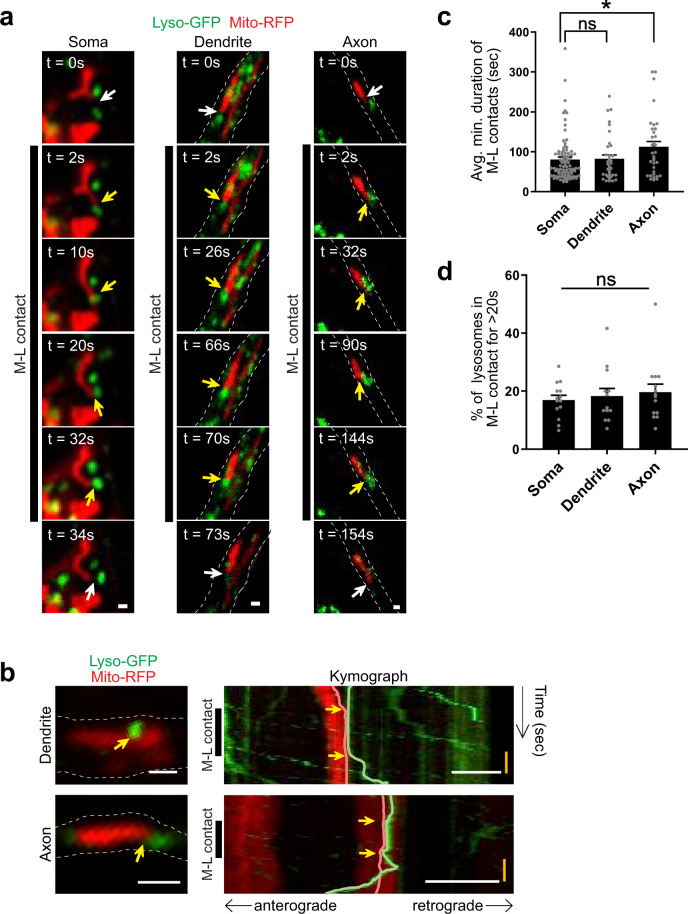
Spatial compartmentalization of neuronal mitochondria–lysosome contact dynamics. **a** Representative time-lapse confocal images of contacts between mitochondria (red, Mito-RFP) and lysosomes (green, Lyso-GFP) in soma, dendrites, and axons of wild-type human dopaminergic iPSC-derived neurons. Time-lapse recordings were taken at 2 s intervals for 5 min. Yellow arrows mark stable M–L contacts. White arrows mark the site of M–L contacts before contact formation or after contact untethering. Black line shows duration of contacts. Scale bar = 500 nm (see Supplementary Movies 4–6). **b** Representative frames of live-cell imaging (left) and dual color kymographs (right) of M–L contacts in dendrites and axons (mitochondria: red, Mito–RFP; lysosomes: green, Lyso-GFP). In confocal image frames (left), contact sites are denoted by yellow arrows. In kymographs: white scale bar = 1 μm, yellow vertical bar = 30 s. Yellow arrows in kymograph point to start and end timepoints of M–L contact tethering. Left black line shows duration of contacts. **c**, **d** Quantification of M–L contacts across different neuronal compartments. One-way ANOVA followed by Tukey’s multiple comparisons test. **c** Comparison of average minimum durations of M–L contacts in soma, dendrites, and axons (*n* = 86 (soma), *n* = 35 (dendrite), *n* = 34 (axon) contacts from 26 neurons, *N* = 3 independent experiments), *p = 0.0335. **d** Percentage of lysosomes contacting mitochondria in soma, dendrites, and axons (for >20 s) (*n* = 14 (soma), *n* = 13 (dendrite), *n* = 14 (axon) neurons; *N* = 3 independent experiments). For all quantifications, data are means ± S.E.M; *p ≤ 0.05, ns: not significant.

Next, we investigated the spatial compartmentalization of neuronal M–L contacts in the soma, dendrites, and axons of human neurons, as both organelles are localized throughout multiple neuronal compartments. Dendrites and axons were identified as being positive for MAP2 or Tau, respectively (Supplementary Fig. 2c). Importantly, we found that mitochondria and lysosomes tethered at contact sites (yellow arrows) within all three neuronal compartments in the soma (Fig. 2a (left) and Supplemental Movie 4), dendrites (Fig. 2a (middle), Supplemental Movie 5), and axons (Fig. 2a (right) and Supplemental Movie 6). M–L contacts in dendrites and axons exhibited decreased mitochondrial and lysosomal motility (yellow arrows) (Fig. 2b). To further examine whether the dynamics of contacts differed by spatial compartmentalization, we quantified the duration of M–L contacts and percentage of lysosomes in contact with mitochondria across different neuronal compartments. Interestingly, live-cell imaging analysis revealed that the average minimum duration of M–L contacts was significantly increased in axons compared to those in the soma (soma: 80.6 ± 6.2 s; dendrites: 82.4 ± 9.7 s; axons: 112.5 ± 13.2 s) (Fig. 2c). In contrast, the percentage of lysosomes contacting mitochondria in the soma, dendrites, and axons did not differ (Fig. 2d). Together, these results demonstrate that M–L contacts are able to form with varying dynamics across multiple neuronal compartments in human neurons.

### Loss of GCase activity disrupts M–L contact untethering in *GBA1*-PD patient dopaminergic neurons

PD has been genetically and functionally linked to both mitochondrial and lysosomal defects^9,10^, but whether M–L contacts are disrupted in PD has not been previously investigated. As mutations in the lysosomal enzyme GCase (*GBA1*) represent the greatest genetic risk factor for PD^12^, and GCase activity is decreased in both idiopathic and multiple types of familial PD patient neurons^9,16–18^, we examined M–L contact dynamics in *GBA1*-PD (Δhet 84GG) patient neurons.

Using PD patient fibroblasts harboring mutant *GBA1*, we generated human iPSCs and their isogenic controls by CRISPR-Cas9^16^ (Supplementary Fig. 1a) which were subsequently differentiated into midbrain dopaminergic neurons (mutant *GBA1* (∆GBA); isogenic control (Corr)) (Supplementary Fig. 2a–c). Both mutant *GBA1* and its isogenic control did not affect the efficiencies of fibroblast reprogramming to iPSCs or differentiation into midbrain dopaminergic neurons from iPSCs^16^.

**Fig. 3:**
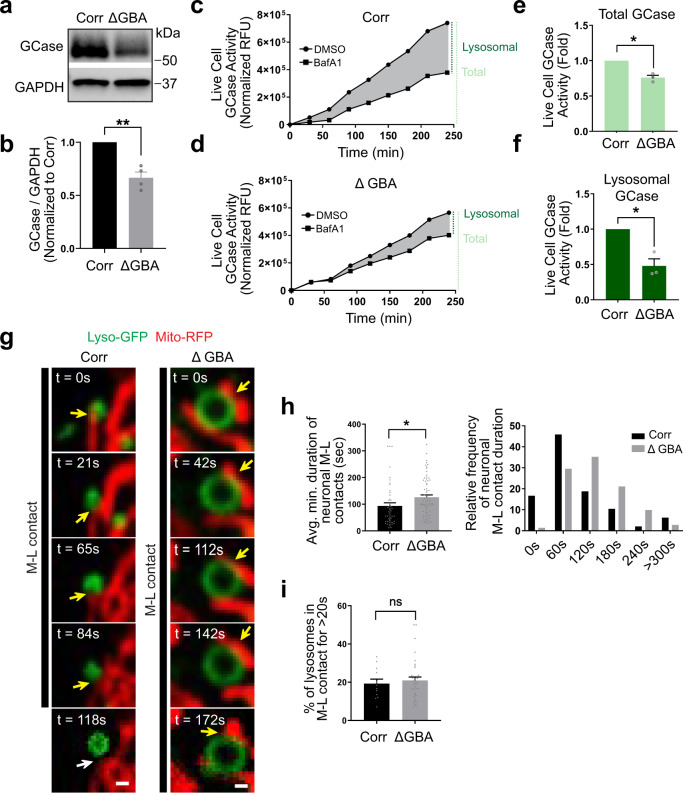
Loss of GCase activity disrupts mitochondria–lysosome contact untethering in *GBA1*-PD patient dopaminergic neurons. **a**, **b** Western blot analysis of PD patient-derived mutant *GBA1* dopaminergic neurons (∆GBA; het 84GG) and its CRISPR-corrected isogenic control (Corr) neurons. GCase level was significantly reduced in ∆GBA neurons (*N* = 4 independent experiments). Paired two-sided Student’s *t*-test; **p = 0.0083. **c**–**f** ∆GBA and Corr neurons were treated with either DMSO or BafA1 and subjected to live-cell GCase activity analysis. **e** Quantification of the area under each curve (AUC) demonstrates decreased total GCase activity in ∆GBA neurons. Paired two-sided Student’s *t*-test; *p = 0.0183. **f** Lysosomal GCase activity was calculated by subtracting BafA1 values from DMSO. Values are expressed as fold-change compared to isogenic controls (*N* = 3 independent experiments). Paired two-sided Student’s *t*-test; *p = 0.0352. **g** Representative time-lapse confocal images of contacts between mitochondria (red, Mito-RFP) and lysosomes (green, Lyso-GFP) in Corr (left) and ∆GBA (right) human neurons. Yellow arrows mark stable M–L contacts. White arrows mark the site of M–L contacts after contact untethering. Black line shows duration of contacts. Scale bar = 500 nm (see Supplementary Movies 7 and 8). **h** Quantification of average minimum duration (left) and relative frequency distribution of the duration of stable M–L contacts (right), showing increased duration of stable M–L contacts in ∆GBA neurons (*n* = 48 contacts from Corr and *n* = 71 contacts from ∆GBA, *N* = 3 independent experiments). *X*-axis of the histogram represents bin centers. Bin width = 60 s. Unpaired two-sided Student’s *t*-test; *p = 0.0204. **i** Percentage of lysosomes contacting mitochondria (for >20 s) (*n* = 12 Corr and *n* = 36 ∆GBA neurons, *N* = 3 independent experiments). Unpaired two-sided Student’s *t*-test. For all quantifications, data are means ± S.E.M.; *p ≤ 0.05, **p ≤ 0.01, ns: not significant.

We first confirmed that PD patient-derived mutant *GBA1* dopaminergic neurons exhibited decreased GCase protein levels (Fig. 3a, b). We also conducted live-cell GCase activity assays and showed that mutant *GBA1* patient neurons had reduced total GCase enzymatic activity compared to isogenic control neurons (Fig. 3c–e). To further compare lysosomal and non-lysosomal GCase activity separately, we used Bafilomycin A1 (BafA1), an inhibitor of lysosomal acidification, as an established protocol for examining lysosomal GCase activity^24^ and also found a significant decrease in lysosomal GCase activity in patient-derived mutant *GBA1* neurons compared to CRISPR-corrected isogenic control neurons (Fig. 3c, d, f), consistent with previous findings^16^.

We next investigated whether lysosomal contacts with mitochondria were disrupted by loss of lysosomal GCase activity by conducting confocal live-cell microscopy of mitochondria and lysosomes in mutant *GBA1* and CRISPR-corrected isogenic control neurons. Interestingly, while M–L contacts dynamically formed in both conditions (yellow arrows) (Fig. 3g and Supplemental Movies 7, 8), the average duration of M–L contact tethering was significantly increased in mutant *GBA1* neurons, indicative of inefficient untethering events (Fig. 3h). However, the percentage of lysosomes in contacts was similar between conditions (Fig. 3i), suggesting that the subsequent untethering but not initial formation of M–L contact tethering was disrupted in mutant *GBA1* neurons.

To further examine M–L contacts, we conducted PLA imaging of M–L contacts in mutant *GBA1* and CRISPR-corrected isogenic control neurons (Supplementary Fig. 4c), and similarly found that M–L contacts could still form in both conditions (Supplementary Fig. 4d). In addition, we conducted EM imaging of M–L contacts in mutant *GBA1* and CRISPR-corrected isogenic control neurons (Supplementary Fig. 4e), and also found that M–L contacts tethered together in both conditions, with the length of membrane contact between mitochondria and lysosomes not altered in mutant *GBA1* neurons (Supplementary Fig. 4f). Of note, ER–mitochondria contact and ER–lysosome contact formation were also not disrupted in mutant *GBA1* neurons (Supplementary Fig. 5a–f). Together, our results suggest that loss of GCase activity does not disrupt M–L contact formation, but preferentially disrupts the untethering of M–L contact sites, resulting in prolonged contact site tethering between mitochondria and lysosomes.

We further examined whether these changes in M–L contact dynamics might be specific to loss of GCase activity rather than general lysosomal enzyme dysfunction. Mutant *GBA1* neurons had altered lysosomal morphology, including enlarged lysosomes (Fig. 3g and Supplementary Fig. 3h, i). However, inhibition of other lysosomal enzymes in human neurons which also led to enlarged lysosomal morphology (Supplementary Fig. 6a) including inhibition of lysosomal acid ceramidase (carmofur treatment), cysteine proteases (E64D treatment), or aspartyl proteases (pepstatin A treatment) did not disrupt M–L contact dynamics (Supplementary Fig. 6b). Thus, our results suggest that this pathway is selectively disrupted by loss of GCase activity rather than general lysosomal defects or enzyme dysfunction.

In addition, we assessed whether rescuing GCase activity in mutant *GBA1* neurons was sufficient to restore defective M–L contact dynamics. The modulator S-181 was recently found to increase GCase activity in mutant *GBA1* neurons^16^. Importantly, S-181 treatment (Supplementary Fig. 6c) rescued the prolonged M–L contact tethering in mutant *GBA1* neurons (Supplementary Fig. 6d), further highlighting the role of GCase activity in regulating M–L contact dynamics.

### Tethering proteins mediating M–L contact are disrupted in *GBA1*-PD patient dopaminergic neurons

**Fig. 4:**
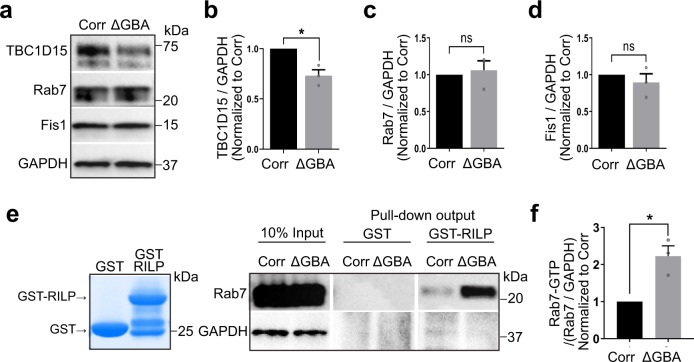
Tethering proteins mediating mitochondria–lysosome contact are disrupted in *GBA1*-PD patient dopaminergic neurons. **a**–**d** Western blot analysis of **b** TBC1D15, **c** Rab7, and **d** Fis1 levels in PD patient-derived mutant *GBA1* dopaminergic neurons (∆GBA) and its CRISPR-corrected isogenic control (Corr) neurons. Protein levels were normalized to loading control GAPDH. Values are expressed as fold-change compared to Corr (*N* = 3 independent experiments). Paired two-sided Student’s *t*-test; *p = 0.0442. **e**, **f** GST-RILP pull-down to measure GTP-bound Rab7 levels in ∆GBA and Corr neurons. **f** Rab7-GTP levels were normalized to total Rab7 normalized to GAPDH. Values are expressed as fold-change compared to Corr (*N* = 3 independent experiments). Paired two-sided Student’s *t*-test; *p = 0.0478. For all quantifications, data are means ± S.E.M., *p ≤ 0.05.

We subsequently examined the potential mechanism through which mutant *GBA1* might disrupt M–L contact dynamics. We recently showed that M–L contact untethering is mechanistically regulated by lysosomal RAB7-GTP hydrolysis from GTP to GDP-bound Rab7^2^, and is driven by the GAP (GTPase-activating protein) activity of mitochondrial TBC1D15 (Rab7 GAP)^3,5^, which is recruited to the outer mitochondrial membrane by Fis1^4,6^. Thus, we investigated whether RAB7-GTP hydrolysis might be disrupted by mutant *GBA1* in patient neurons. We first measured the total protein levels of RAB7 together with Fis1 and TBC1D15 in PD patient-derived mutant *GBA1* dopaminergic neurons compared to isogenic controls (Fig. 4a–d). We observed no differences in total Rab7 levels (Fig. 4c) or Fis1 levels (Fig. 4d), and Fis1 levels were not altered even after normalizing for mitochondrial levels (Fis1/Tom20: Corr vs *GBA1*, *p* = 0.23 (not significant)) (Supplementary Fig. 3j). Of note, TBC1D15 and Fis1 localization to mitochondria (Supplementary Fig. 7a, b), and Rab7 localization to lysosomes (Supplementary Fig. 7c), as well as the levels of the Rab7 GEF (guanine nucleotide exchange factor) complex proteins Mon1 and Ccz1 (Supplementary Fig. 7d) were also not altered in mutant *GBA1* neurons. However, we surprisingly found that TBC1C15 levels were significantly decreased in mutant *GBA1* patient-derived neurons (*p ≤ 0.05) (Fig. 4b).

Based on these findings, we hypothesized that disrupted M–L contact untethering in mutant *GBA1* neurons might be due to defective Rab7-GTP hydrolysis as a consequence of reduced TBC1D15 levels (Rab7 GAP). To test this, we performed GST-RILP (Glutathione Transferase-Rab Interacting Lysosomal Protein) pull-down assays^25^ to measure GTP-bound Rab7 levels, as RILP preferentially binds to GTP-bound Rab7^26,27^ (Fig. 4e). Importantly, we found that PD patient-derived mutant *GBA1* dopaminergic neurons demonstrated significantly increased levels of RAB7-GTP/total Rab7 compared to CRISPR-corrected isogenic control neurons (*p ≤ 0.05) (Fig. 4e, f). Together, these results suggest that decreased TBC1D15 levels in mutant *GBA1* neurons disrupt Rab7 GTP hydrolysis, resulting in increased GTP-bound Rab7 and prolonged M–L contact tethering dynamics.

To further investigate the downregulation of TBC1D15 in mutant *GBA1* neurons, we analyzed TBC1D15 by qPCR and showed that decreased TBC1D15 protein levels in mutant *GBA1* neurons were not due to lower TBC1D15 transcripts levels (Supplementary Fig. 8a). Rather, decreased TBC1D15 protein levels resulted from elevated proteasomal degradation of TBC1D15, which could be inhibited by lactacystin, leading to similar TBC1D15 protein levels between mutant *GBA1* and isogenic control neurons (Supplementary Fig. 8b). We next examined whether mitochondrial dysfunction was sufficient to disrupt TBC1D15 expression levels. Treatment of either the mitochondrial uncoupler carbonyl cyanide p-trifluoromethoxyphenylhydrazone (CCCP), Complex I inhibitor rotenone, Complex III inhibitor Antimycin, or the ATP synthase inhibitor Oligomycin (Supplementary Fig. 8c–f) did not disrupt TBC1D15 expression levels in HeLa cells. In contrast, increasing GCase activity in mutant *GBA1* neurons using the modulator S-181^16^ was sufficient to rescue the decreased levels of TBC1D15 (Supplementary Fig. 6e, f). Thus, these findings suggest that TBC1D15 levels are downregulated at the protein level by proteosomal degradation in mutant *GBA1* neurons, due to the loss of GCase activity rather than general mitochondrial dysfunction.

Finally, we examined if loss of TBC1D15 was able to disrupt M–L contact dynamics in human neurons. Knockdown of TBC1D15 in wild-type iPSC-derived neurons (Supplementary Fig. 9a) increased M–L contact tethering duration (Supplementary Fig. 9b), consistent with findings in non-neuronal cells^2^ and further supporting our results that decreased TBC1D15 levels in PD *GBA1*-linked patient neurons disrupt M–L contact dynamics.

### Inhibition of GCase activity disrupts M–L contact untethering

**Fig. 5:**
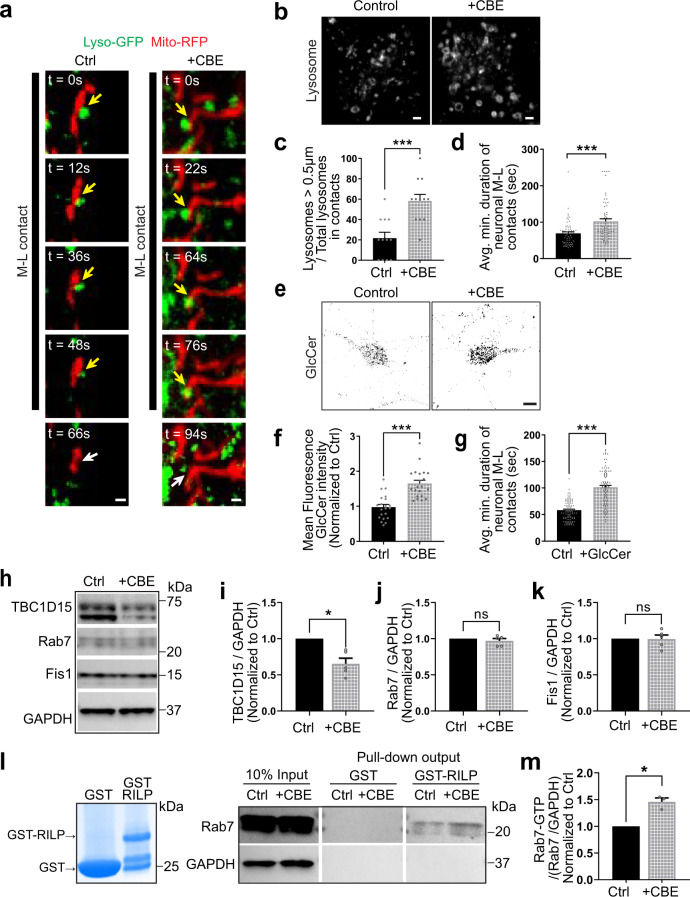
Inhibition of GCase activity disrupts mitochondria–lysosome contact untethering in dopaminergic neurons. **a** Healthy control WT human iPSC-derived dopaminergic neurons were treated with vehicle (Ctrl) or CBE (50 μM; 7 days) to inhibit GCase activity. Representative time-lapse confocal images of contacts between mitochondria (red, Mito-RFP) and lysosomes (green, Lyso-GFP) in Ctrl (left) and CBE-treated (right) human neurons. Yellow arrows mark stable M–L contacts. White arrows mark the site of M–L contacts after contact untethering. Black line shows duration of contacts. Scale bar = 500 nm. **b** Representative confocal images of lysosomes (LAMP1–GFP) in the Ctrl (left) and CBE-treated (right) human neurons. Scale bar = 1 μm. **c** The effects of CBE treatment on lysosomal morphology were confirmed by quantification of the percentage of lysosomes that were enlarged (diameter >0.5 μm) in M–L contacts (*n* = 12 neurons per condition, *N* = 3 independent experiments). Unpaired two-sided Student’s *t*-test; ***p = 0.0001. **d** CBE treatment in control neurons increased the average minimum duration of stable M–L contacts (*n* = 60 contacts per condition, *N* = 3 independent experiments). Unpaired two-sided Student’s *t*-test; ***p = 0.0001. **e** Representative confocal images of immunocytochemistry of GlcCer in the Ctrl (left) and CBE-treated (right) human neurons. Scale bar = 5 μm. **f** Quantification of CBE treatment leading to increased GlcCer levels as measured by immunofluorescence signal of GlcCer (*n* = 21 neurons per condition, *N* = 3 independent experiments). **g** Exogenous GlcCer treatment in control neurons increased the average minimum duration of stable M–L contacts (*n* = 93 contacts from Ctrl, *n* = 94 contacts from +GlcCer neurons, *N* = 3 independent experiments). **f**, **g** Unpaired two-sided Student’s *t*-test; ***p < 0.0001. **h**–**k** Western blot analysis of **i** TBC1D15, **j** Rab7, and **k** Fis1 levels in Ctrl and CBE-treated neurons. Protein levels were normalized to loading control GAPDH. Values are expressed as fold-change compared to Ctrl (*N* = 5 independent experiments). Paired two-sided Student’s *t*-test; *p = 0.0104. **l**, **m** GST-RILP pull-down to measure GTP-bound Rab7 levels in Ctrl and CBE-treated neurons. **m** Rab7-GTP levels were normalized to total Rab7 normalized to GAPDH. Values are expressed as fold-change compared to Ctrl (*N* = 3 independent experiments). Paired two-sided Student’s *t*-test; *p = 0.0242. For all quantifications, data are means ± S.E.M., *p ≤ 0.05, ***p ≤ 0.001.

To further examine whether M–L contact untethering defects were indeed dependent on loss of GCase activity, we treated wild-type human midbrain dopaminergic neurons expressing wild-type GCase with the GCase inhibitor conduritol-b-epoxide (CBE) (50 μM; 7 days) which decreases neuronal GCase activity and promotes GlcCer accumulation^14,16,28^. Using confocal live-cell time-lapse imaging of mitochondria and lysosomes in both wild-type (Ctrl) and CBE-treated (+CBE) human neurons, we found that M–L contacts dynamically formed in both conditions (yellow arrows) (Fig. 5a). In addition, CBE treatment was effective in increasing lysosomal size as has been previously reported^29^ (Fig. 5b, c). Importantly, we found that inhibition of GCase activity by CBE treatment resulted in significantly prolonged M–L contact tethering duration (***p ≤ 0.001) (Fig. 5a, d), consistent with what we observed in mutant *GBA1* patient-derived neurons, and further confirming the effect of decreased GCase activity on disrupting M–L contact untethering.

We additionally validated these findings in two other wild-type iPSC-derived neuronal lines which we characterized for iPSC and midbrain dopaminergic neuron-specific markers (Supplementary Fig. 10a–c). Inhibition of GCase activity with CBE treatment in both lines also led to significantly prolonged M–L contact tethering duration (Supplementary Fig. 10d, e), further supporting our results that inhibition of GCase activity disrupts M–L contact dynamics in human neurons.

As loss of GCase activity results in significantly increased GlcCer levels (Fig. 5e, f), we then asked whether treatment of exogenous GlcCer in wild-type iPSC-derived neurons could be sufficient to disrupt M–L contact dynamics. Using live-cell microscopy of mitochondria and lysosomes in wild-type iPSC-derived neurons treated with exogenous GlcCer, we found that this also led to significantly prolonged contact site tethering duration (***p < 0.001) (Fig. 5g), highlighting a role for increased GlcCer levels in mutant *GBA1* neurons in dysregulating M–L contacts.

Next, we investigated whether inhibition of GCase activity also disrupted M–L contact untethering machinery. We measured the total protein levels of Rab7 together with TBC1D15 and Fis1 in wild-type (Ctrl) and CBE-treated (+CBE) humans neurons (Fig. 5h–k). Indeed, CBE-treated neurons also showed significantly decreased TBC1C15 levels (*p ≤ 0.05) (Fig. 5h, i), without changes in total Rab7 or Fis1 levels (Fig. 5j, k), consistent with mutant *GBA1* patient-derived neurons. To further support these findings, wild-type iPSC-derived neurons treated with exogenous GlcCer also showed reduced TBC1D15 levels (Supplementary Fig. 10f, g).

We then examined whether inhibition of GCase activity by CBE treatment led to similar defects in Rab7 GTP hydrolysis, resulting in elevated GTP-bound Rab7 levels. Using the GST-RILP pull-down assay in CBE-treated neurons (Fig. 5l), we further found that GCase inhibition resulted in increased levels of RAB7-GTP/total Rab7 (Fig. 5l, m), consistent with what we observed in mutant *GBA1* neurons. In summary, our results from both mutant *GBA1* and CBE-treated neurons support the hypothesis that loss of GCase activity, which increases GlcCer levels, leads to disruption of TBC1D15 levels and Rab7-GTP hydrolysis in neurons, resulting in the misregulation of M–L contact untethering dynamics.

### Mitochondrial dysfunction due to prolonged M–L contacts is partially rescued by TBC1D15 expression

**Fig. 6:**
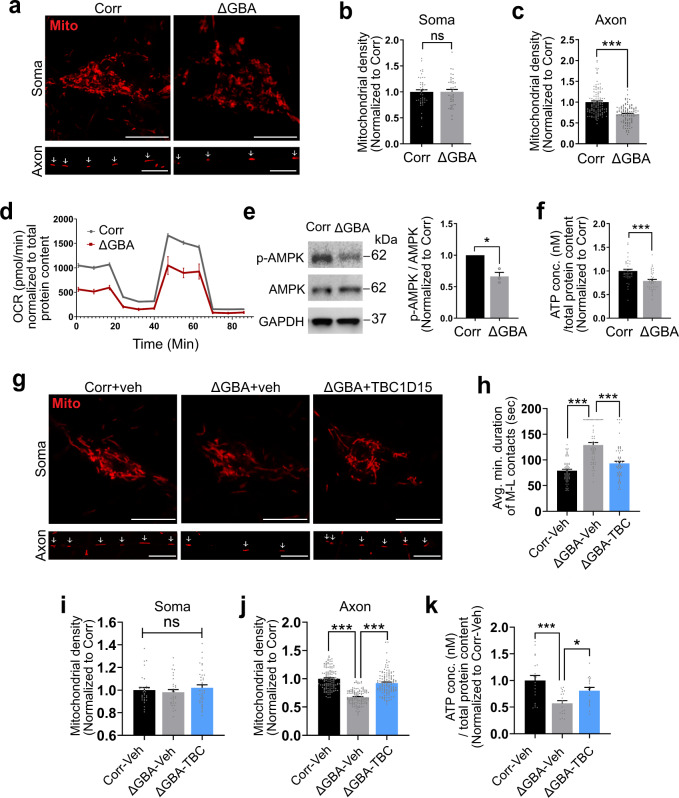
Mitochondrial dysfunction due to prolonged mitochondria–lysosome contacts is partially rescued by expression of TBC1D15 in *GBA1*-PD patient dopaminergic neurons. **a** Live-cell distribution of mitochondria (TMRM, red) in the soma and axons from PD patient-derived mutant *GBA1* dopaminergic neurons (∆GBA) and its CRISPR-corrected isogenic control (Corr) neurons. White arrows mark mitochondria in a single axon. Scale bar, 10 μm. **b** Quantified mitochondrial density in the soma (TMRM-positive pixels/pixels of soma). Corr (*n* = 47 neurons), ∆GBA (*n* = 47 neurons) (*N* = 3 independent experiments). **c** Quantified mitochondrial density in axons (mitochondria count/length of axon (μm)). Corr (*n* = 122 axons), ∆GBA (*n* = 114 axons) (*N* = 3 independent experiments). **b**, **c** Unpaired two-sided Student’s *t*-test; ***p < 0.0001. **d** Oxygen consumption rate (OCR) was measured and normalized to total protein content. Corr (*n* = 9 samples), ∆GBA (*n* = 9 samples) (*N* = 3 independent experiments). **e** Western blot analysis of phospho-AMPKα and AMPKα levels in Corr and ∆GBA neurons. Ratio of p-AMPKα/AMPKα are expressed as fold-change compared to Corr (*N* = 3 independent experiments). Paired two-sided Student’s *t*-test; *p = 0.0354. **f** Total cellular ATP content was measured and normalized to total protein content (ng/μl). Corr (*n* = 40 samples), ∆GBA (*n* = 40 samples) (*N* = 3 independent experiments). Unpaired two-sided Student’s *t*-test; ***p < 0.0001. **g** Live-cell distribution of mitochondria (TMRM, red) in the soma and axons from Corr and ∆GBA human neurons with or without human TBC1D15 lentiviral expression. White arrows mark mitochondria in a single axon. Scale bar, 10 μm. **h** Quantification of average minimum duration of M–L contacts in Corr-vehicle (*n* = 62 contacts from 18 neurons), ∆GBA-vehicle (*n* = 60 contacts from 15 neurons), ∆GBA-TBC1D15 (*n* = 63 contacts from 19 neurons) (*N* = 3 independent experiments); ***p < 0.0001. **i** Quantified mitochondrial density in the soma (TMRM-positive pixels/pixels of soma). Corr-vehicle (*n* = 32 neurons), ∆GBA-vehicle (*n* = 31 neurons), ∆GBA-TBC1D15 (*n* = 38 neurons) (*N* = 3 independent experiments). **j** Quantified mitochondrial density in axons (mitochondria count/length of axon (μm)). Corr-vehicle (*n* = 124 axons), ∆GBA-vehicle (*n* = 127 axons), ∆GBA-TBC1D15 (*n* = 127 axons) (*N* = 3 independent experiments), ***p < 0.0001. **k** Total cellular ATP concentration was measured and normalized to total protein content (ng/μl). Corr-vehicle (*n* = 19 samples), ∆GBA-vehicle (*n* = 19 samples), ∆GBA-TBC1D15 (*n* = 19 samples) (*N* = 3 independent experiments), ***p = 0.0002, *p = 0.0493. **h**–**k** One-way ANOVA followed by Tukey’s multiple comparisons test. For all quantifications, data are the means ± S.E.M.; *p ≤ 0.05, ***p ≤ 0.001, ns: not significant.

M–L contacts mediate the bidirectional regulation of both mitochondrial and lysosomal network dynamics, and importantly are able to directly regulate both mitochondrial dynamics and motility^2,30–32^. We thus investigated whether mitochondrial dynamics might be disrupted in PD patient-derived mutant *GBA1* dopaminergic neurons, consistent with defective M–L contact untethering, by analyzing the distribution of mitochondria in the soma and axons. Healthy mitochondria with intact mitochondrial membrane potential were imaged by live-cell imaging in patient-derived mutant *GBA1* (∆GBA) and CRISPR-corrected isogenic control (Corr) neurons (Supplementary Fig. 3c). Interestingly, while there was no difference in mitochondrial density in the soma (Fig. 6a, b and Supplementary Fig. 3k), we found that axonal mitochondrial density was significantly decreased in mutant *GBA1* neurons compared to axons of isogenic control neurons (Fig. 6a, c), suggesting that mutant *GBA1* preferentially disrupts axonal mitochondrial distribution in Parkinson’s patient neurons. In addition, we also observed defective mitochondrial respiration as measured by decreased oxygen consumption rate (OCR) in mutant *GBA1* neurons (Fig. 6d), as well as decreased AMPK activation (Fig. 6e), compared to isogenic control neurons. We also measured cellular ATP level and found significantly decreased ATP levels in mutant *GBA1* neurons compared to isogenic controls (Fig. 6f), even after normalization for mitochondrial mass (***p < 0.001*;* Corr vs *GBA* [ATP intensity/TOM20 levels]). Consistent with our findings that TBC1D15 levels were decreased in mutant *GBA1* neurons, we also observed mitochondrial dysfunction in TBC1D15 knockdown neurons (Supplementary Fig. 9c).

Based on these findings, we hypothesized that dysregulated M–L contact untethering dynamics in mutant *GBA1* neurons might contribute to these defects in mitochondrial distribution and function. Thus, to test if these defects could be rescued by promoting M–L contact untethering in mutant *GBA1* neurons, we expressed human TBC1D15 in mutant *GBA1* neurons by lentiviral transduction (Fig. 6g and Supplementary Fig. 11a). The expression of exogenous TBC1D15 in neurons was confirmed by western blot analysis and fluorescence imaging (Supplementary Fig. 11b, c). Importantly, consistent with our previous findings that TBC1D15 promotes M–L contact untethering^2^, transduction of wild-type TBC1D15 in mutant *GBA1* neurons (∆GBA+TBC1D15) was able to promote M–L contact untethering, resulting in significantly decreased M–L contact durations compared to lentiviral vehicle-treated neurons (∆GBA+veh) (***p ≤ 0.001) (Fig. 6h).

We further investigated the effect of rescuing M–L contact dynamics on axonal mitochondrial density in mutant *GBA1* neurons. Interestingly, while TBC1D15 expression did not alter mitochondrial densities in the soma of mutant *GBA1* neurons (Fig. 6g, i), it significantly rescued the decreased mitochondrial density in axons of mutant *GBA1* neurons (Fig. 6g, j). Moreover, TBC1D15 expression was also able to partially rescue ATP levels in mutant *GBA1* neurons (Fig. 6k). Together, our results suggest that upregulation of TBC1D15 in PD patient mutant *GBA1* neurons is sufficient to rescue the misregulation of M–L contact untethering, as well as downstream defects in mitochondrial dynamics and function.

Finally, we extended our analysis to additional PD mutant *GBA1* patient neurons (Δhet N370S) and CRISPR-edited isogenic control neurons (Supplementary Fig. 12a–c). Mutant *GBA1* (N370S) patient neurons also showed decreased GCase levels and enzymatic activity (Supplementary Fig. 12d–g). Importantly, they also had significantly increased GTP-bound Rab7 due to lower TBC1D15 levels (Supplementary Fig. 12h–m), which resulted in prolonged M–L contact tethering (Supplementary Fig. 12n). Moreover, S-181 modulator treatment in mutant *GBA1* (N370S) neurons was sufficient to rescue both M–L contact dynamics (Supplementary Fig. 12n) as well as TBC1D15 levels (Supplementary Fig. 12o). In addition, mutant *GBA1* (N370S) neurons also demonstrated reduced mitochondrial function (Supplementary Fig. 12p). Thus, these findings further highlight the role of defective GCase activity in PD *GBA1-*linked patient neurons on disrupting Rab7 GTP hydrolysis machinery and M–L contact site dynamics.

## Discussion

Our study demonstrates that M–L contact sites dynamically form in human neurons, and further investigates their role in neurons from patients with *GBA1*-linked PD. We found that loss of lysosomal GCase enzymatic activity in PD patient-derived dopaminergic neurons led to prolonged M–L contact tethering dynamics due to defective contact untethering machinery, and resulted in misregulated axonal distribution of mitochondria and decreased ATP levels. Importantly, we showed that rescuing M–L contact site dynamics in PD patient neurons is sufficient to ameliorate defects in mitochondrial distribution and function, thus highlighting a potential role for M–L contact site dysregulation in PD pathogenesis.

Multiple genes linked to mitochondria or lysosomes have been identified as causative or risk genes of PD^33,34^. Moreover, both mitochondrial and lysosomal dysfunction have been implicated in PD^9,10,15,35–40^, suggesting a functional crosstalk between these two organelles. We previously found that oxidized dopamine mediates convergence of mitochondrial and lysosomal dysfunction in human but not in mouse dopaminergic neurons^9^, highlighting the importance of patient-derived dopaminergic neurons for studies of PD pathogenesis.

Despite the previously studied role of mitochondria and lysosomes in PD pathogenesis, a direct homeostatic relationship between these two organelles that is independent of eventual lysosomal degradation of mitochondria has not been examined. In this context, our work provides evidence for the role of M–L contacts not only in the homeostasis of dopaminergic neurons but also as a link between mitochondrial and lysosomal dysfunction in PD pathogenesis. We specifically focused on *GBA1* mutations which represent the greatest genetic risk factor for PD^11,12^. Importantly, wild-type GCase enzyme activity is also reduced in patient neurons with genetic or idiopathic PD who do not harbor *GBA1* mutations^9,16,18^, suggesting that loss of GCase activity is an important contributor to PD pathogenesis. Previously, we reported that the loss of GCase function in patient neurons compromises lysosomal protein degradation^15^ which contributes to other key PD pathogenic phenotypes including α-synuclein accumulation^13,15,24,41^. In addition to its primary lysosomal dysfunction, *GBA1* mutations and abnormal GCase activity have also been linked to mitochondrial dysfunction^9,28,42–46^.

In this study, we show that *GBA1* mutant PD patient neurons have defective M–L contact dynamics, resulting in prolonged contact tethering. While previous studies did not examine the GTP-bound state of Rab7^47,48^, we found that loss of GCase activity resulted in an increased percentage of active GTP-bound lysosomal Rab7, which directly mediates M–L contact tethering^2^. This is likely due to decreased levels of TBC1D15 (Rab7 GAP) in mutant *GBA1* patient neurons, as TBC1D15’s GAP activity promotes Rab7 GTP hydrolysis and subsequent M–L contact untethering^2^. Indeed, we found that increasing TBC1D15 expression was sufficient to rescue the prolonged M–L contact tethering we observed in mutant *GBA1* neurons. In addition, we further observed mitochondrial dysfunction in patient neurons, including abnormal mitochondrial distribution in axons, decreased mitochondrial respiration and lower ATP levels. Of note, TBC1D15 expression was also able to rescue mitochondrial dysfunction. Thus, dysregulation of M–L contacts may play an important role in *GBA1*-linked PD pathogenesis, and targeting contact machinery may help ameliorate downstream mitochondrial dysfunction. Given that mitochondria play key roles as energy suppliers especially in the synapses of active neurons, we hypothesize that such abnormal distribution of mitochondria may further contribute to synaptic dysfunction in PD^10^.

While inter-organelle contact sites have been found to be essential subdomains for modulating cellular function and homeostasis^49–51^, only recently have studies reported the function and molecular architecture of inter-organelle contacts in neurons, such as those between the mitochondria and endoplasmic reticulum^40,52,53^. In addition, dysfunction of inter-organelle contacts in disease have been shown to be key contributors in the development of various diseases^1,30,31,40,54–60^. Moreover, the recent identification of M–L contact sites has shed new light on the direct relationship between mitochondria and lysosomes in a pathway independent of lysosomal degradation of mitochondria^2,31,61–64^, allowing for direct crosstalk and regulation of both organelles in a dynamic manner^1^. In our previous study, we showed the formation of M–L contacts in non-neuronal cells and identified protein mediators responsible for contact untethering^1,2^. Here, we demonstrate that M–L contacts are also key contributors in human neurons and that contacts dynamically form in multiple neuronal compartments, suggesting that they act as important sites for the neuronal regulation of mitochondrial and lysosomal dynamics. Together, our findings not only provide insights into inter-organelle contacts in maintaining the cellular homeostasis of human neurons, but also suggest the importance of M–L contacts as a potential target for therapeutic development in PD.

## Methods

### Human iPSC culture and characterization

Detailed procedures for iPSC culture and neuronal differentiation have been described previously^16^. Healthy control and PD patient skin fibroblasts^16^ (*GBA1* heterozygous 84GG mutation (c.84dupG frameshift mutation) which prevents the expression of the mutant allele, resulting in reduced GCase levels arising from a single wild-type copy of *GBA1;* and *GBA1* heterozygous p.N370S mutation) were reprogrammed into iPSCs through Northwestern University Stem Cell Core Facility, using Sendai virus (SeV)-based delivery of four Yamanaka factors (Oct, Sox2, Klf4, and c-Myc). We also generated an isogenic control iPSC line by correcting the mutation using CRISPR/Cas9 protocols^65^ as recently described^16^. Guide RNAs targeting the mutation were cloned into vector PX461 (Addgene #48140) carrying the cDNA encoding for GFP-tagged Cas9 nuclease. The plasmid was electroporated into *GBA1* 84GG mutant iPSCs together with ssODN carrying the corrected sequence. After 48 h, generation of an isogenic control line was confirmed by FACS-sorting and sequencing. All iPSCs were maintained either in mTeSR™1 or mTeSR™ Plus media (Stemcell Technologies, #85850, #05825) and cells were passaged as small chunks every 6–8 days depending on confluence. All iPSC lines have been routinely characterized for expression of pluripotency markers. Cells were plated on PDL-coated coverslips and subjected to immunofluorescence staining for NANOG, OCT4, SSEA4, and TRA1-81. Genomic integrity was confirmed as described previously^16^. Mycoplasma tests were performed on a monthly basis to maintain qualified iPSC lines.

### Generation and characterization of hiPSC-derived dopaminergic neurons

Human dopaminergic iPSC-derived neurons were differentiated using previously established protocol^22^ for analysis of M–L contacts. Briefly, after plating single iPSCs, cells were treated with factors according to the original protocol. At day 13 of differentiation, cells were passaged en bloc (size of 1–2 mm) onto 10-cm culture dishes pre-coated with poly-d-lysine (PDL) (Sigma, #P1149) / laminin (Invitrogen, #23017-015). At day 25, neurons were treated with accutase and passaged onto PDL/laminin-coated culture dishes, and subjected to dopaminergic marker characterization through immunocytochemistry (ICC) using TH, FOXA2, LMX1A together with neural specific marker β-III-tubulin (TUJ1) at day 30. After day 50, neurons were considered mature and maintained in Neurobasal media (Life Technologies, # 21103049) containing Neurocult SM1 (Stemcell Technologies, #5711). Neurons at day 50–60 were used for immunostaining, EM, live-cell time-lapse confocal imaging, live-cell or fixed-cell super-resolution SIM. Neurons from *N* ≥ 3 independent experiments (biological replicates; batches of neuron differentiation) per condition were used for each experiment.

### Immunocytochemistry

Cells were immunostained for multiple antibodies depending on the purpose of each experiment. Neurons were plated on PDL/laminin-coated coverslips and fixed in 4% paraformaldehyde in PBS for 20 min and permeablized with 10% FBS and 0.1% saponin in PBS at room temperature. For GlcCer staining, 10% FBS was substituted with 2% gelatin (Sigma, #G1393). Cells were then immuno-labeled with the following primary antibodies: Oct4 (Abcam, #19857, 1:100); SSEA1 (Millipore, MAB#4304, 1:100); Nanog (R&D systems, #AF1997, 1:50); Tra-1-81 (Millipore, #MAB4381, 1:100); TH (Calbiochem, #657012, 1:500); β-III-tubulin (Biolegend, #801202, #802001, 1:1000); Lmx1a (Milipore, MAB#10533, 1:500); FoxA2 (Santa Cruz, #sc-101060, 1:100); Tom20 (Abcam, #78547, 1:100); Lamp1 (Santa Cruz, #sc-20011, 1:100); Map2 (Novus Biological, #NB300213, 1:3000); Tau (DAKO, #A002401-2, 1:300); GlcCer (Glycobiotech, #RAS_0011, 1:100). After overnight incubation at 4 °C, coverslips were washed three times with PBS for 5 min each, incubated in Alexa-conjugated secondary antibodies (Invitrogen, #A21206, #A21202, #A11029, #A11011, #A10042, #A10037, #A21449, 1:1000) for 1 h at room temperature, washed three times and mounted onto Superfrost Plus microscope slides (Fisherbrand, #12-550-15) with VECTASHIELD HardSet Antifade Mounting Medium (Vector Labs, #H-1400). Alternatively, ProLong™ Diamond Antifade Mountant (Thermo Fisher Scientific, #P36959) was used for SIM sample preparation. Images were obtained on either a Leica DMI4000B confocal microscope using Leica Application Suite X (Leica) or a Nikon A1R laser scanning confocal microscope with GaAsp detectors using NIS-Elements (Nikon). Colocalization was measured using the EzColocalization plugin in ImageJ (National Institutes of Health (NIH))^66^.

### Proximity ligation assay

Proximity between outer mitochondrial membrane protein Tom20 and lysosomal membrane protein Lamp1 was detected using Duolink™ PLA kit (Sigma Aldrich, #DUO92101) according to the manufacturer’s protocol. Neurons were plated on PDL/laminin-coated coverslips and cells were immuno-labeled with Tom20 (Abcam, #78547, 1:100) and Lamp1 (Santa Cruz, #sc-20011, 1:100). Images of red PLA signals were collected using Nikon A1R laser scanning confocal microscope with GaAsp detectors.

### Electron microscopy

For EM, neurons were grown on PDL/laminin-coated glass coverslips. Neurons were fixed with 2.5% glutaraldehyde and 2% paraformaldehyde in 0.1 M cacodylate buffer, pH 7.3 for 30 min at room temperature, and then submitted to the Northwestern EM core facility for subsequent processing. After post-fixation in 1% osmium tetroxide and 3% uranyl acetate in PBS, cells were dehydrated in an ethanol series, embedded in Epon resin and polymerized for 48 h at 60 °C. Ultrathin sections were made using a UCT ultramicrotome (Leica Microsystems) and contrasted with 4% uranyl acetate and Reynolds’s lead citrate. Samples were imaged on a FEI Tecnai Spirit G2 transmission electron microscope (FEI) operated at 80 kV. Images were captured with an Eagle 4k HR 200kV CCD camera and analyzed using ImageJ (NIH).

### Live-cell time-lapse confocal microscopy

Live-cell time-lapse confocal imaging was conducted of mitochondria and lysosomes labeled with the following reagents: Lentivirus expressing Tom20-RFP or Lamp1-GFP (MOI = 3, 3 days); CellLight^®^ BacMam 2.0 baculovirus (PPC (particles per cell) = 40, 2 days) [Thermo Fisher: Lysosomes-GFP (#C10596), Lysosomes-RFP (#C10597), Mitochondria-RFP (# C10601), ER-GFP (#C10590)]; LysoTracker™ Red DND-99 (2 μM, 45 min; Thermo Fisher, #L7528); MitoTracker™ Green FM (0.125 μM, 45 min; Thermo Fisher, #M7514); and TMRM (0.1 μM, 45 min; Fisher Scientific, #T669), which is a cell-permeant dye that accumulates in active mitochondria with intact membrane potential (ΔΨm). For analysis of lysosomal-drug-treated cultures, neurons were treated with GCase inhibitor CBE (50 μM, 7 days) (Cayman Chemicals, #15216), carmofur (7.5 μM, 2 days) (Cayman Chemicals, #14243), E-64D (10 μM, 24 h) (Cayman Chemicals, #13533), or Pepstatin A (10 μM, 24 h) (Milipore, #516481). For live imaging, neurons were grown on glass-bottomed culture dishes (MatTek, #P35G-1.5-14-C) in Neurobasal media (Life Technologies, #21103049) containing Neurocult SM1 (Stemcell Technologies, #5711). Samples were imaged on a Nikon A1R laser scanning confocal microscope with GaAsP detectors using a Plan Apo λ 100 × 1.45 NA oil immersion objective (Nikon) using NIS-Elements (Nikon). During the imaging, culture dishes were kept in a temperature-controlled chamber (37 °C) at 5% CO_2_.

### Structured illumination microscopy

M–L contact sites in both fixed and live neurons were imaged using super-resolution SIM. For SIM analysis, neurons were cultured on nitric-acid-treated, PDL/laminin-coated High Precision Glass Cover Slip (Bioscience Tools, #CSHP-No1.5-12). Samples were prepared using the same protocol for regular ICC. Super-resolution images were taken on a Nikon N-SIM system with a 100× oil immersion objective lens, 1.49 NA (Nikon). Images were captured and reconstructed using Nikon NIS-Elements.

### Neuronal compartmentalization of M–L contacts

Dendrites and axons were identified as positive for either MAP2 or Tau immunostaining, respectively, in fixed cells. In live-cell imaging, axons and dendrites were distinguished using Map2-GFP lentiviral expression. Neuron cultures had dendrites that were thicker and shorter than axons, with many axons branching from dendrites, consistent with previous observations in dopaminergic neurons^67^. Using these characteristic morphologies, neurites were further characterized as dendrites in subsequent imaging experiments involving live or fixed neurons.

### Image analysis

To quantify contacts, live neurons were imaged at 2-s intervals for 3–6 min. Contacts were defined as mitochondria and lysosomes in close proximity at the beginning of the video and which lasted for greater than 20 s. The duration, number of contacts, and the density of mitochondria were analyzed manually in NIS-Elements (Nikon) for further examination. The minimum duration of contacts was quantified as the time before contact termination and dissociation (mitochondria and lysosomes detaching from one another) over the course of 5-min videos. Any contacts that lasted beyond 5 min were categorized as 300 s in bar graphs and as >300 s in histograms for the minimum duration of M–L contacts. The percentage of lysosomes in contacts was quantified as the percentage of lysosomes in contact with mitochondria for greater than 20 s divided by the total number of Lyso-GFP-positive vesicles in the region of interest. The length of neurites and the area of cell bodies were measured using a built-in function of ImageJ (NIH).

### Generation and transduction of lentiviral constructs

Human MAP2 Lentiviral cDNA ORF vector was purchased from Sino Biological Inc. (#HG13690-ACGLN). Human TBC1D15 ORF in the plasmid generated by Vector Builder was subjected to Q5^®^ Site-Directed Mutagenesis (New England Biolabs, #E0554S) to insert NheI enzyme cutting site (5ʹ-GCTAGC-3ʹ) and then cloned into lentiviral vector including BFP tag (Addgene, #117136). mApple-Tom20 (Addgene, #54955) and LAMP1-mGFP (Addgene, #34831) were obtained from Addgene plasmids using the NheI/NotI cut sites and subcloned into pER4 lentiviral expression vector. For TBC1D15 knockdown, shRNA constructs against TBC1D15 were obtained from Horizon Discovery (#RHS4533-EG64786) together with TRC Lentiviral Non-targeting shRNA Control (#RHS6848). To generate the lentivirus control, the nuclear localization signal (NLS) of SV40 large T antigen was introduced after BFP coding sequence in the same lentiviral plasmid (Addgene #117136) used for hTBC1D15 cloning. Lentiviral vectors were packaged and transfected into HEK 293FT cells using X-treme Gene HP DNA transfection (Roche, #06366236001) together with helper plasmids psPAX2 (Addgene, #12260) and pLP3 (Invitrogen). Quantitation of retroviral antigens was determined using ZeptoMetrix Corporation RETROtek HIV-1 p24 Antigen ELISA kits (Fisher Scientific, #22-156-700). Concentrated viruses were aliquoted and kept at −80 °C for future use. For Map2 expression, neurons were transduced with Map2 Lentivirus and incubated 4 days before live-cell confocal imaging or fixation. For TBC1D15 expression, neurons at day 35 were transduced with hTBC1D15 Lentivirus, incubated 15 days (MOI = 8) until day 50 and were subjected to further analysis. Expression of constructs delivered by lentiviral infection was verified by either immunoblot analysis or imaging of the fluorescent label.

### Live-cell GCase activity assay

The enzymatic activity of GCase in live human neurons was measured as previously described^15^ with minor modifications. The neuron culture media was changed to phenol red-free Neurobasal media (Thermo Fisher, #12348017) containing Neurocult SM1 (Stemcell Technologies, #5711) a day before the measurement. The next day, half of the neurons were treated with DMSO and 50 μg/ml fluorescein di-β-d-glucopyranoside (PFB-FDGluc) (Fisher Scientific, #P11947) in phenol red-free Neurobasal media. Another half were treated with 400 nM BafA1 (Cayman chemicals, #11038) and 50 μg/ml PFB-FDGluc in phenol red-free Neurobasal media. Neurons were incubated for 1 h at 37 °C in the dark to allow for the accumulation of PFB-FDGluc, a substrate of GCase, in lysosomes. GCase activity was quantified for 4.5 h with 30 min intervals by measuring fluorescence upon the cleavage of PFB-FDGluc over time in a Spectramax i3 plate reader (Molecular Devices) (Ex = 485 nm, Em = 525 nm). Activity within the lysosomal compartment was determined by measuring the response to BafA1. Non-lysosomal GCase activity was interpreted as the activity that was not responsive to BafA1. After the last measurement, neurons were washed three times with PBS, and were lysed in RIPA lysis buffer. BCA protein assay was done according to the manufacturer’s protocol (Thermo Fisher, #23225) to measure the total protein amount required for normalization. Total GCase activity was quantified by calculating the area under the DMSO curve (AUC) using Prism7 (GraphPad) software. Lysosomal GCase activity was obtained by measuring the area between BafA1 and DMSO curves.

### SDS-PAGE and Western blotting

Neurons were collected in ice-cold PBS and centrifuged at 400×*g* for 5 min at specific time points (e.g. day 30 for neuron characterization, day 40–70 for biochemical analysis of organelle contacts). Pellets were lysed in N-PER™ Neuronal Protein Extraction Reagent (Thermo Scientific, #87792) with cOmplete protease inhibitor cocktail (Sigma, #11836170001), and lysates were collected according to the manufacturer’s protocol. After boiling for 20 min in 4× Laemmeli sample buffer, protein samples were separated on 4–20% Tris-glycine precasted gel (Invitrogen, #XP04202BOX) and transferred to PVDF or nitrocellulose membranes using Trans-blot TurboTM transfer system (Biorad). Membranes were blocked with 5% milk in 1× Tris-buffered saline (50 mM Tris, pH 7.4, 150 mM NaCl) with 0.1% Tween (TBST) for 1 h at room temperature and incubated with a primary antibody, 4 °C, overnight: Rab7 (Cell Signaling, #D95F2, 1:1000; Abcam, #ab137029, 1:1000); TBC1D15 (Sigma Aldrich, #SAB2701508, 1:500); Fis1 (Alexis, #ALX-210-1037-0100, 1:1000); GAPDH (Millipore, #MAB374, 1:2000); GBA (Abnova, #H00002629-M01, 1:500); TH (Calbiochem, #657012, 1:1000); Synapsin (Santa Cruz, #sc-398849, 1:1000); β-III-tubulin (TUJ1) (Biolegend, #801202, 1:5000); AMPKα (Cell Signaling, #2532, 1:1000); Phospho-AMPKα (Cell Signaling, #2535, 1:1000); Ccz1 (Santa Cruz, #sc-514290, 1:1000); Mon1 (Abcam, #ab103919, 1:500); Tom20 (Abcam, #ab56783, 1:1000); Lamp1 (Santa Cruz, #sc-20011, 1:500). The next day, after three times of washing with 1× TBST, membranes were incubated in secondary goat anti-mouse and goat anti-rabbit HRP antibody (Jackson Immuno Research Lab, #115-035-146, #111-035-144, 1:10,000) diluted in 5% milk in 1× TBST for 1 h and washed three times with 1× TBST. HRP signal was developed using Clarity chemiluminescence substrate (Biorad, #170-5061) or Lumigen ECL Ultra (Lumigen, #TMA-100), and images were taken on the ChemiDoc XRS+ imaging station (Biorad). Protein levels were normalized against GAPDH. Quantification was done using ImageJ (NIH).

### Quantitative RT-PCR

Total RNA was purified from neurons using RNeasy Micro Kit (Qiagen, #74004) and transcribed to cDNA using the High-Capacity cDNA Reverse Transcription Kit (Thermo Fisher, #4368814). Quantitative RT-PCR was performed using the 7500 Fast Real-Time PCR system (Applied Biosystems), with ssoAdvanced universal SYBR Green Supermix (BioRad, #1725271). The following pre-designed primer sets were used: TBC1D15 (PrimerBank ID #226342866c3) and GAPDH (PrimerBank ID #378404907c2) (Supplementary Table 1).

### GST-RILP pull-down assay

To determine active GTP-bound Rab7 levels in neurons, GST-RILP pull-down assay was performed as previously described^25^ with minor modifications. Plasmids were gifts from Aimee Edinger (Addgene plasmid #79149). GST-control and GST-fused Rab7 binding domain of RILP protein (nucleotides 658–897) were expressed in BL21 bacteria. Bacteria were collected and lysed in B-PER™ Bacterial Protein Extraction Reagent (Thermo Scientific, #78248) with 1,4-Dithiothreitol (DTT, 1 mM), EDTA (1 mM), and cOmplete protease inhibitor cocktail (Sigma, #11836170001). Proteins were purified using pre-equilibrated 50% slurry of glutathione-Sepharose 4B beads (GE Healthcare) and quantified using the BCA assay. Neurons to be analyzed in the pull-down assay were pelleted and lysed in pull-down buffer (20 mM HEPES, 100 mM NaCl, 5 mM MgCl_2_, 1% TritonX-100, and protease inhibitors). GST-control beads and GST-RILP beads were added to the neuron lysates and the samples were rocked overnight at 4 °C, followed by washing with cold pull-down buffer. Bound proteins were eluted by boiling in 2× Laemmli sample buffer (Sigma Aldrich, #S3401) and used for western blot analysis.

### Seahorse XF Cell Mito Stress Test

OCR of neurons was analyzed using an XF 24 Extracellular Flux Analyzer (Seahorse Biosciences) according to the manufacturer’s protocol; 1.25 × 10^5^ neurons were plated on one well of XF24 cell culture microplates. Four empty wells without neurons were used as background control for temperature-sensitive fluctuations in OCR analysis. Before the assay, culture medium was replaced with Seahorse XF medium (Seahorse Bioscience, #103575-100) supplemented with 1 mM sodium pyruvate (Corning^®^, #25-000-CI), 10 mM d-glucose (Sigma Aldrich, #G8769), and 2 mM glutamine (Gibco, #25030-081) and incubated for 1 h in a CO_2_-free incubator. OCR was measured, after sequential injection of 1 µM oligomycin, CCCP, and Antimycin A. After the assay, neurons were lysed and subjected to BCA protein assay (Thermo Fisher, #23225) for normalization.

### Total cellular ATP assay

Total cellular ATP content was measured using the ATPlite kit (PerkinElmer, #6016943) according to the manufacturer’s protocol. Neurons were plated on black 96-well plates (Thermo Fisher, #237108) at a density of 5 × 10^4^/well. Neurons were lysed in lysis solution provided in the kit and luminescence was measured in a Spectramax i3 plate reader (Molecular Devices). Neuron lysates were subjected to BCA protein assay (Thermo Fisher, #23225) for normalization.

### Mitochondrial drug treatment

HeLa cells were cultured in Dulbecco’s modified Eagle’s medium (DMEM) (Gibco, #11995-065) supplemented with 10% FBS, 100 U/ml Penicillin–Streptomycin (Gibco, #15140-122). A day after passaging, cells were treated for 0, 4, and 8 h with CCCP (10 μM) (Sigma Aldrich, #C2759), Rotenone (100 nM) (Sigma Aldrich, #R8875), Antimycin (1 μM) (Sigma Aldrich, #A8674), or Oligomycin (1 μM) (Sigma Aldrich, #O4876). Treated cells were lysed in RIPA buffer (Boston Bioproduct, #BP-115-5x) with cOmplete protease inhibitor cocktail (Sigma, #11836170001), and lysates were subjected to BCA protein assay (Thermo Fisher, #23225) and SDS-PAGE.

### Statistical analysis

For all statistical tests, cells from *N* ≥ 3 independent experiments (biological replicates) per condition were used (see text and figure legends for details). Data were analyzed using unpaired two-tailed Student’s *t*-test (for two datasets) or one-way ANOVA with Tukey’s post-hoc test (for multiple datasets). Bar graphs presented are in the form of means ± SEM. Statistics and graphing were performed using Prism 7 (GraphPad) software. Videos and images were processed using NIS-Elements (Nikon) and assembled using ImageJ. All figures were assembled in Adobe Illustrator.

### Reporting summary

Further information on research design is available in the Nature Research Reporting Summary linked to this article.

## Supplementary information

Supplementary Information

Description of Additional Supplementary Files

Supplementary Movie 1

Supplementary Movie 2

Supplementary Movie 3

Supplementary Movie 4

Supplementary Movie 5

Supplementary Movie 6

Supplementary Movie 7

Supplementary Movie 8

Reporting Summary

## Data Availability

All data that support the findings of this study are included in the manuscript or are available from the authors upon reasonable request. Source data are provided with this paper.

## Source data

Source Data
