# Supplementary material for: Dysregulation of mitochondria-lysosome contacts by GBA1 dysfunction in dopaminergic neuronal models of Parkinson’s disease
Source: Nat Commun. 2021 Mar 22;12:1807. doi: 10.1038/s41467-021-22113-3 (PMC7985376; doi:10.1038/s41467-021-22113-3)
Supplement: Supplementary file 1 — Supplementary Information [file 41467_2021_22113_MOESM1_ESM.pdf]

Supplementary Information

Dysregulation of mitochondria-lysosome contacts by GBA1 dysfunction in dopaminergic neuronal models of Parkinson's disease

Soojin Kim[1], Yvette C. Wong[1], Fanding Gao[1], Dimitri Krainc[1*]

[1]Department of Neurology, Northwestern University Feinberg School of Medicine, 303 E Chicago Avenue, Chicago, IL, 60611, USA

*Correspondence: dkrainc@nm.org

Supplementary Table 1. Primer sequences

	Primer Bank ID	Forward primer sequence	Reverse primer sequence
**TBC1D15**	226342866c3	AAAAGGACCCTTATACGGCAAC	CGCTGCCTCTCAAACTGTCAA
**GAPDH**	378404907c2	ACAACTTTGGTATCGTGGAAGG	GCCATCACGCCACAGTTTC

Supplemental Figures

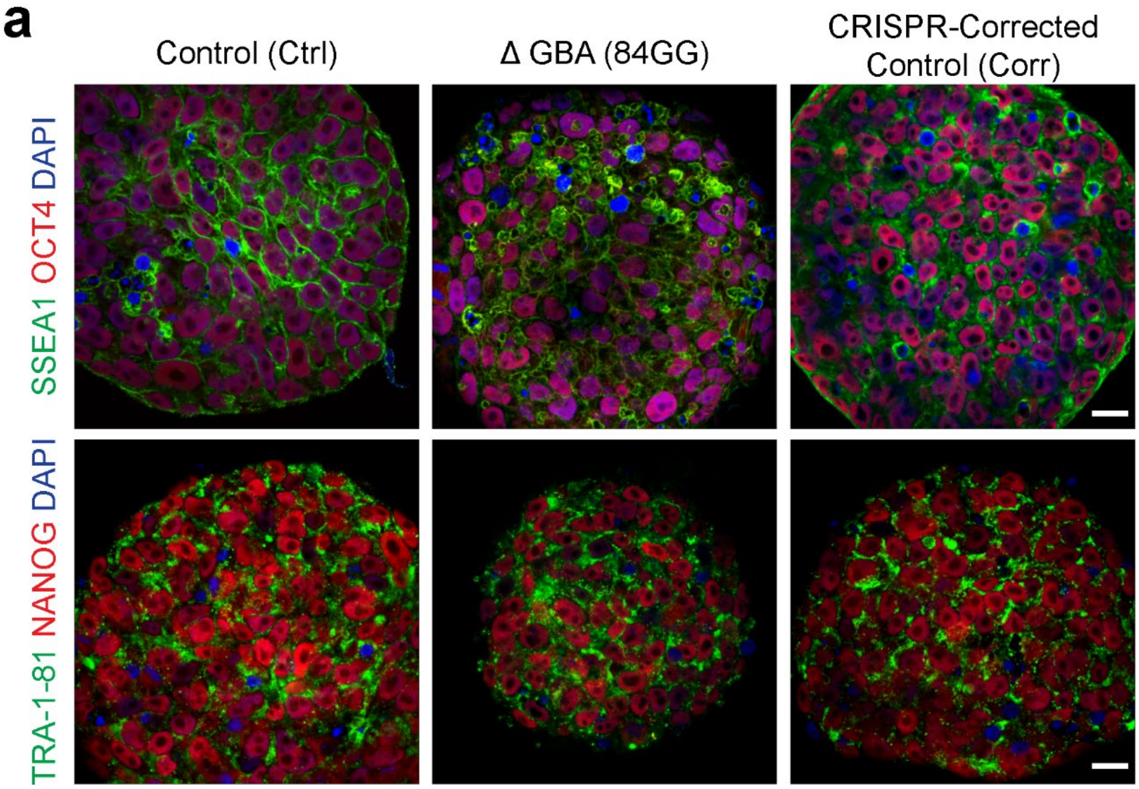

Supplementary Fig.1 Characterization of wild-type control, PD patient mutant *GBA1* and CRISPR-corrected isogenic control human iPSCs (induced pluripotent stem cells).

a Immunocytochemistry demonstrated that wild-type Control (Ctrl), Mutant *GBA*1 (ΔGBA; het 84GG) and CRISPR-corrected isogenic control (Corr) human induced Pluripotent Stem Cells (iPSCs) expressed pluripotency markers including Oct4, SSEA1, TRA-1-81 and Nanog. Top panel, merged images of Oct4 (red), SSEA1 (green), and DAPI (blue) nuclear staining from each lines. Bottom panel, merged images of Tra 1-81 (green), Nanog (red), and DAPI (blue) nuclear staining. Scale bar, 10μm. (N = 3 independent experiments).

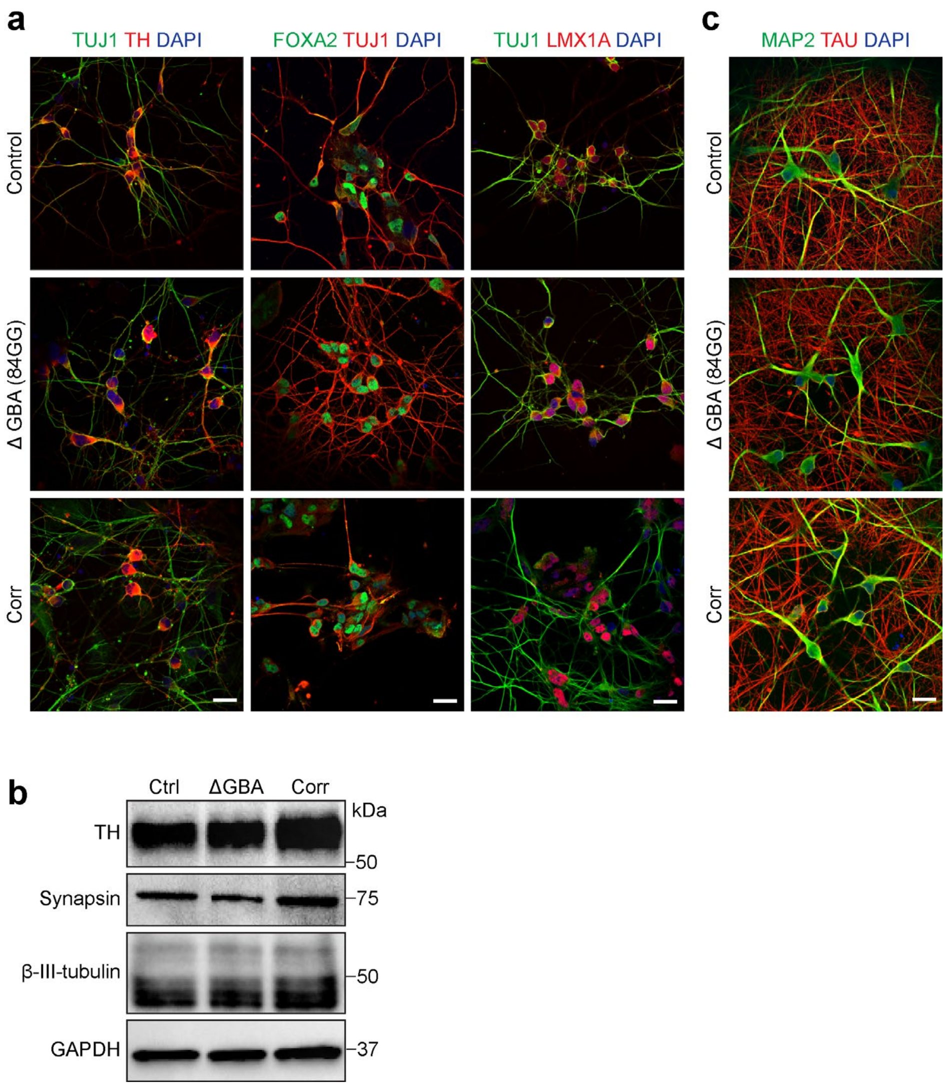

Supplementary Fig.2 Characterization of wild-type control, PD patient mutant *GBA1* and CRISPR-corrected isogenic control human iPSC-derived dopaminergic neurons.

a Human iPSCs from wild-type control (Control), mutant *GBA1* (ΔGBA) and CRISPR-corrected isogenic control (Corr) lines were differentiated into midbrain dopaminergic neurons. Neurons were analyzed by immunofluorescence for the expression of dopamine (TH; tyrosine hydroxylase), midbrain (FOXA2, LMX1A) and neuronal cytoskeletal (Tuj1) markers at day 30. Scale bar = 20µm. **b** Western blot analysis of human iPSC-derived dopaminergic neurons at day 50. The expression of dopaminergic neuronal markers (TH, Synapsin and β−iii Tubulin) were confirmed in Ctrl, ΔGBA and Corr neurons. GAPDH was used as a loading control. (N = 3 independent experiments). **c** Immunocytochemistry of dendritic marker Map2 (green) and axonal marker tau (red) in human iPSC-derived dopaminergic neurons at day 50. Scale bar = 20µm. (N = 3 independent experiments).

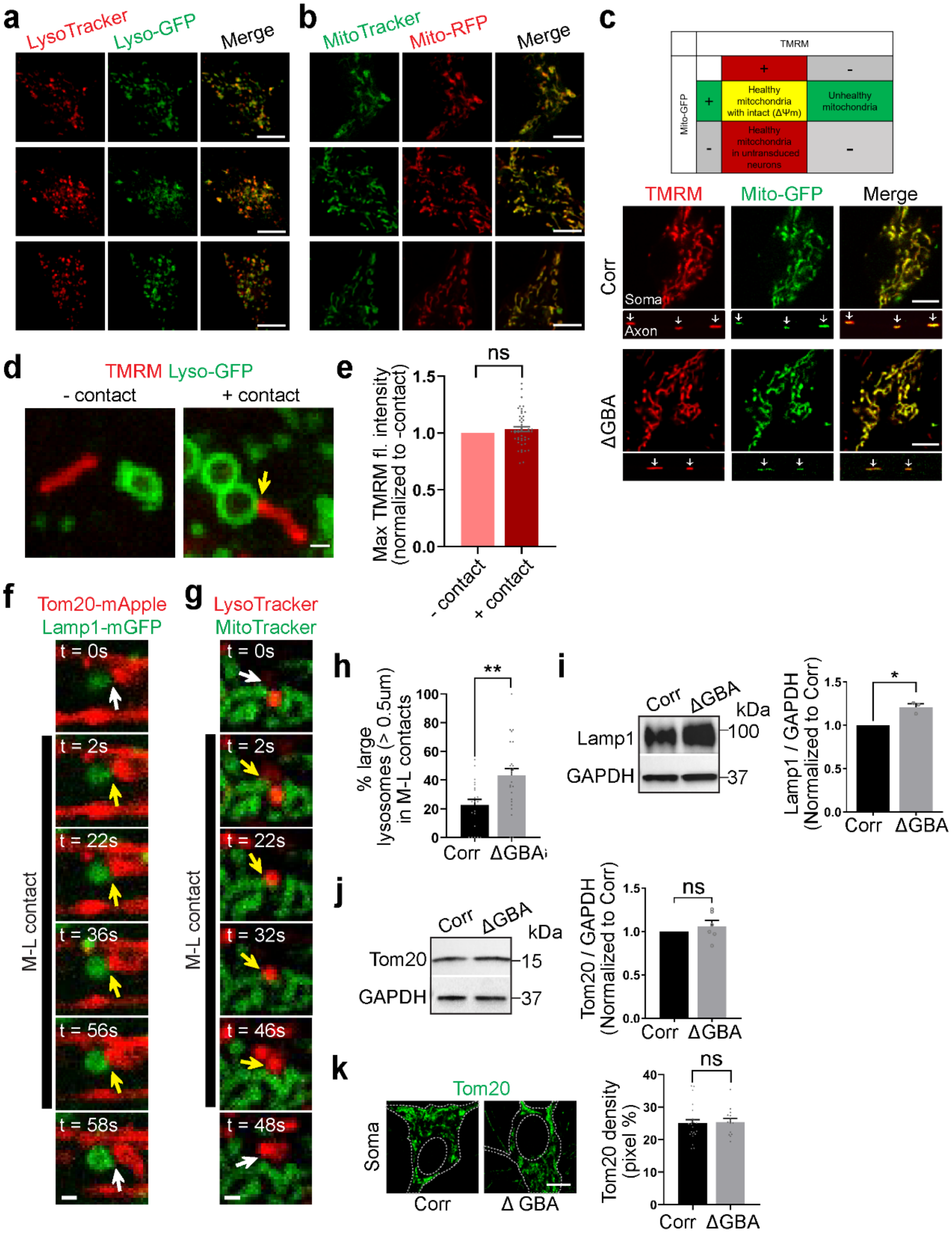

Supplementary Fig.3 Additional characterization of mitochondria-lysosome contact sites in human dopaminergic neurons.

a Representative fluorescence confocal live-cell images of lysosomal markers in wild-type human iPSC-derived dopaminergic neurons showing CellLight® Lysosome-GFP (Lyso-GFP, green) colocalized with LysoTracker™ Red DND-99. (N = 3 independent experiments). **b** Representative fluorescence confocal live-cell images of mitochondrial markers in wild-type neurons showing CellLight® Mitochondria-RFP (Mito-RFP, red) colocalized with MitoTracker Green FM. (N = 3 independent experiments). **c**

Tetramethylrhodamine ethyl ester (TMRM, red) accumulates in active mitochondria with intact membrane potentials (ΔΨm), and colocalized with CellLight® Mitochondria-GFP (Mito-GFP, green) which labels all mitochondria in transduced neurons (table). Representative confocal images of mitochondria labelled with TMRM and/or Mito-GFP in PD patient-derived mutant *GBA1* dopaminergic neurons (ΔGBA) and its CRISPR-corrected isogenic control (Corr) neurons. Arrows mark mitochondria in a single axon. Scale bar, 5µm. (N = 3 independent experiments) **d** Representative live-cell images of TMRM signal in mitochondria that are not in contact (left; - contact) or in contact with a lysosome (right; + contact). Yellow arrow marks stable M-L contacts. Scale bar = 500nm. **e** Quantification of maximum TMRM fluorescence intensity in mitochondria showing that it is similar for mitochondria that are not in contact (- contact) compared to mitochondria in contact with a lysosome (+ contact). (50 mitochondria per condition from 26 neurons, N = 3 independent experiments). Paired two-sided Student's t-test. **f-g** M-L contacts were visualized by multiple methods for labeling mitochondria and lysosomes: Representative time-lapse confocal images of M-L contacts between **f** outer mitochondrial membrane fluorescent marker (red, Tom20-mApple) and lysosomal membrane fluorescent marker (green, Lamp1-mGFP) or **g** LysoTracker (red) and MitoTracker (green) in control neurons. Yellow arrows mark stable M-L contacts. White arrows mark the site of M-L contacts before and after contacts. Black line shows duration of contacts. Scale bar = 500nm. **h** Quantification of the percentage of large lysosomes (diameter >0.5um) in M-L contacts in Corr and ΔGBA neurons. Unpaired two-sided Student's t-test; **p=0.0016. **i-j** Western blot analysis of **i** Lamp1 (N = 3 independent experiments) and **j** Tom20 (N = 6 independent experiments) in Corr and ΔGBA neurons. GAPDH was used as a loading control. Values are expressed as fold-change compared to Corr. Paired two-sided Student's t-test; *p=0.0324. **k** Immunocytochemistry of mitochondrial marker Tom20 (green) in soma. The density of signal was calculated as the percentage of green pixels divided by the total number of pixels in a neuron. (n = 24 Corr neurons and n = 15 ΔGBA neurons from N = 3 independent experiments). Unpaired two-sided Student's t-test.

For all quantifications, data are the means ± S.E.M. *p≤ 0.05, **p≤0.01, ns: not significant.

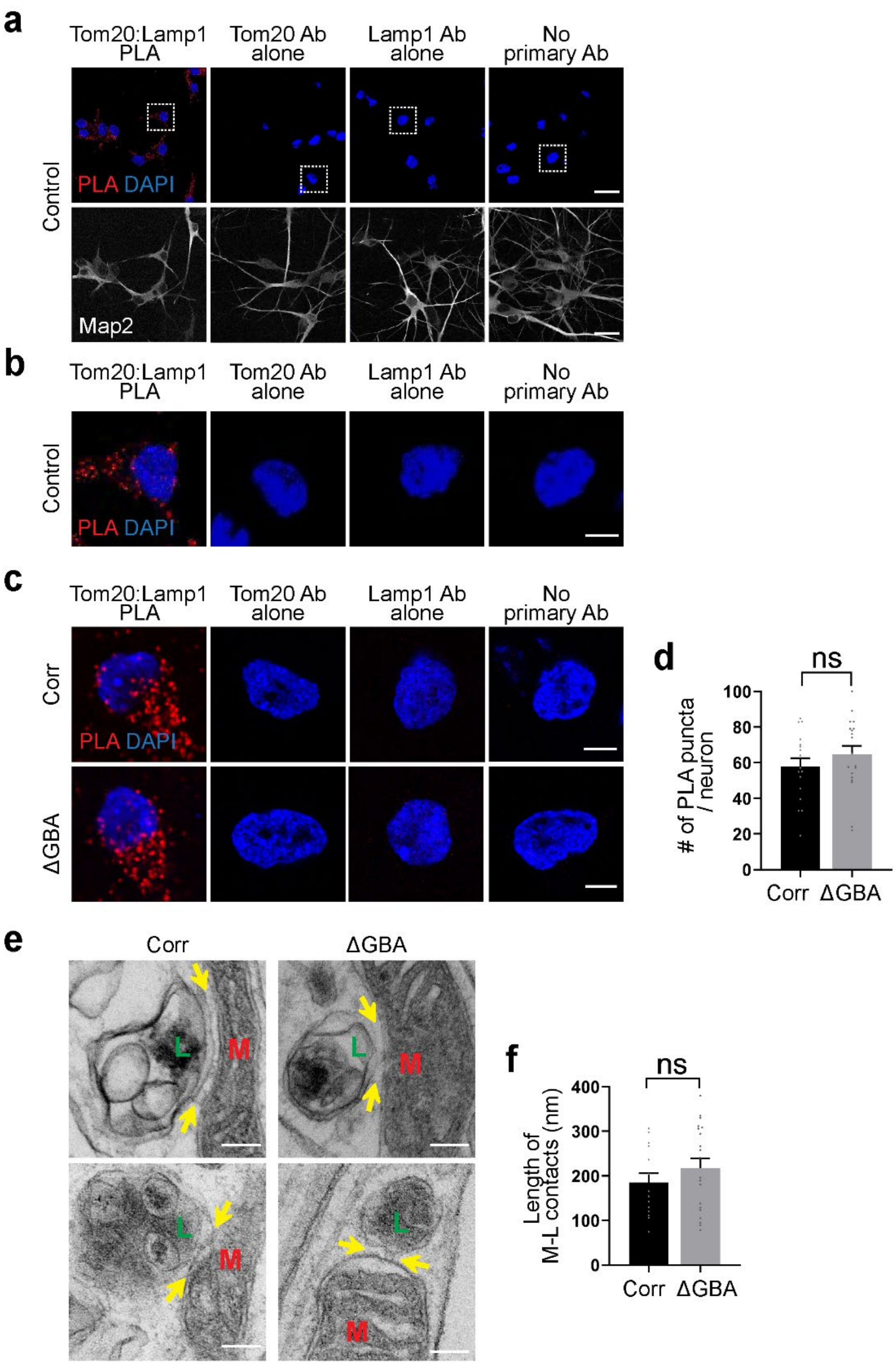

Supplementary Fig.4 Proximity ligation assay (PLA) and electron microscopy of mitochondria-lysosome contact sites in human dopaminergic neurons.

a-c Representative confocal images of mitochondria-lysosome contact sites in human iPSC-derived dopaminergic neurons using imaging of *in situ* proximity ligation assay (red) for endogenous Tom20 and Lamp1 in (**a**; inset in **b**) wild-type control (N = 3 independent experiments) and **c** PD patient-derived mutant *GBA1* dopaminergic neurons (ΔGBA) and its CRISPR-corrected isogenic control (Corr) neurons.

Neurons immunostained with only Tom20 or Lamp1, or without primary antibody were used as negative controls. The nucleus is stained with DAPI (blue). Scale bar = (**a**) 20µm, (**b-c**) 5µm. **d** Quantification of the number of PLA puncta in Corr and ΔGBA neurons (n = 31 Corr neurons, 23 ΔGBA neurons, N = 3 independent experiments). **e-f** Representative electron microscopy (EM) images of M-L contacts (yellow arrows) with distance between membranes <10nm (mitochondria, M; lysosomes, L) in Corr and ΔGBA neurons. **f** Quantification of the length of contacts (membrane contact distance) (n = 34 contacts from 27 neurons, N = 3 independent experiments). **e** Scale bar = 100nm. **d,f** Unpaired two-sided Student's t-test. For all quantifications, data are the means ± S.E.M. , ns: not significant.

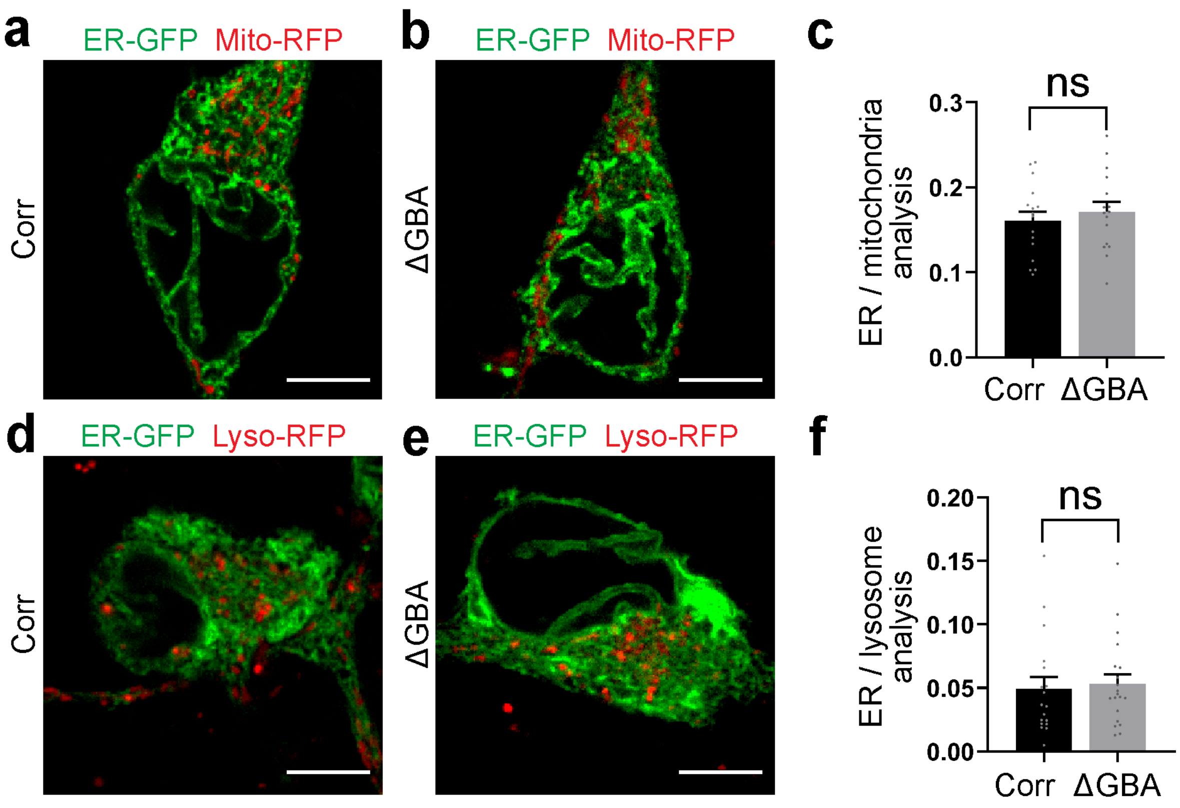

Supplementary Fig.5 ER-mitochondria and ER-lysosome contact site analysis in *GBA1*-PD patient dopaminergic neurons.

a-b Representative confocal images of ER-mitochondria contacts (ER: green, ER-GFP; mitochondria: red, Mito-RFP) in soma of PD patient-derived mutant *GBA1* dopaminergic neurons (ΔGBA) and its CRISPR-corrected isogenic control (Corr) neurons. **c** Mander's overlap coefficient (MOC) values measured from each frame of 150 sec time-lapse imaging movie were averaged to quantify the fraction of ER overlapped with mitochondria. (n = 16 Corr neurons, 16 ΔGBA neurons, N = 3 independent experiments). **d-e** Representative confocal images of ER-lysosome contacts (ER: green, ER-GFP; lysosome: red, Lyso-RFP) in Corr and ΔGBA neurons. **f** MOC values measured from each frame of 150 sec time-lapse imaging movies were averaged to quantify the fraction of ER overlapped with lysosomes. (n = 18 Corr neurons, 20 ΔGBA neurons, N = 3 independent experiments). **c,f** Unpaired two-sided Student's t-test.
For all quantifications, data are the means ± S.E.M. ns: not significant. Scale bar = 5µm.

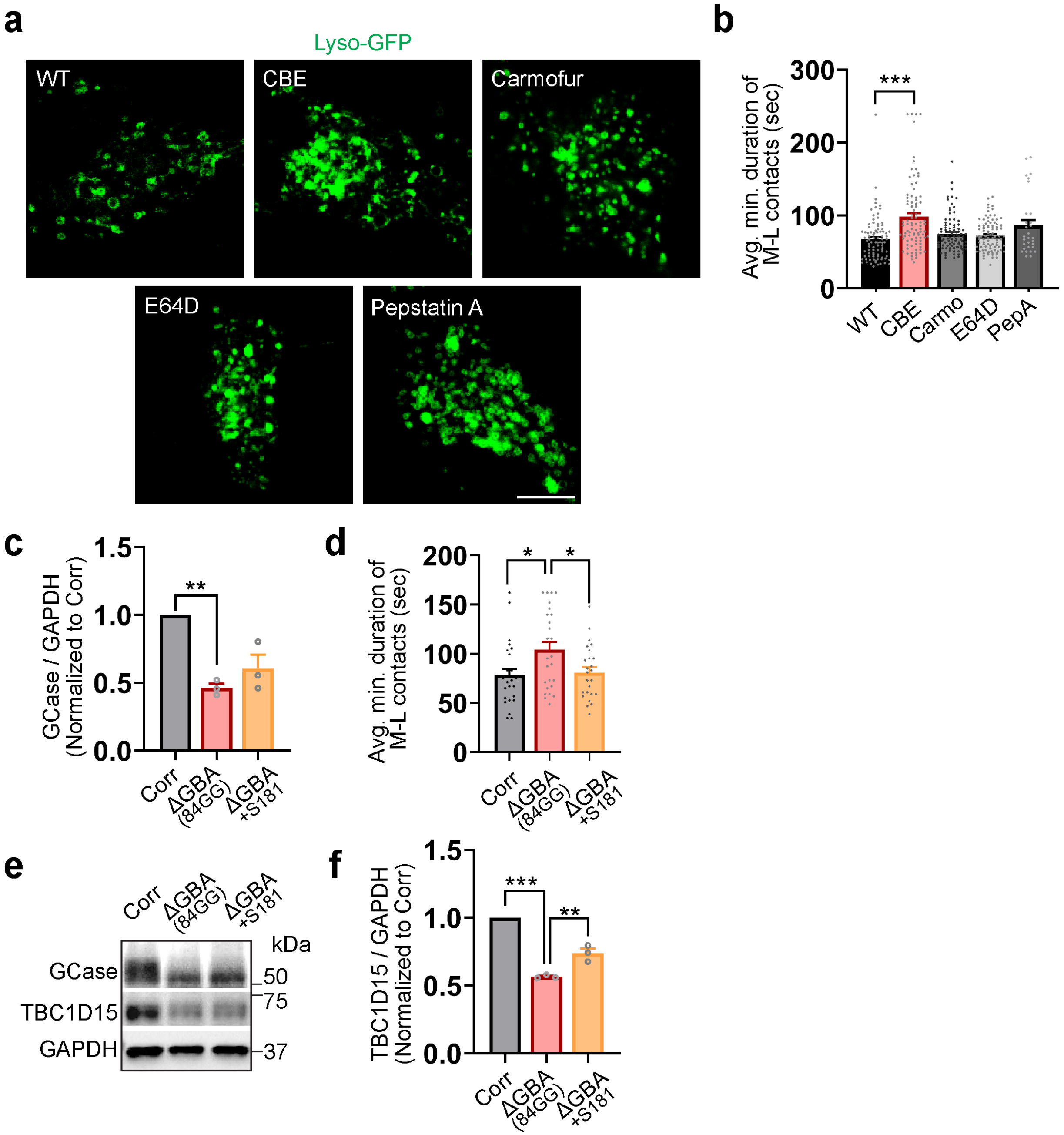

Supplementary Fig.6 Lysosomal dysfunction specifically from loss of GCase activity disrupts mitochondria-lysosome contact dynamics.

a Representative confocal images of lysosomes in wild-type human iPSC-derived dopaminergic neurons treated with lysosomal enzyme inhibitors: CBE (inhibitor of GCase); Carmofur (inhibitor of acid ceramidase); E64D (inhibitor of cysteine proteases); Pepstatin A (inhibitor of aspartyl proteases). Scale bar = 5µm. **b** CBE treatment in wild-type neurons increased the average minimum duration of stable M-L contacts compared to untreated neurons (WT), while other lysosomal enzyme inhibitors did not. (WT (n = 90 contacts from 18 neurons), CBE (n = 90 contacts from 18 neurons), Carmo (n = 92 contacts from 19 neurons), E64D (n = 91 contacts from 20 neurons), PepA (n = 30 contacts from 6 neurons), N = 4 independent experiments). One-way ANOVA followed by Tukey's multiple comparisons test;

p<0.0001. **c, e, f** Western blot analysis of (**c**) GCase (**p=0.0021) and (**f**) TBC1D15 (p<0.0001; **p=0.0024) in PD patient-derived mutant *GBA1* dopaminergic neurons (ΔGBA) and its CRISPR-corrected isogenic control (Corr) neurons, and S181-treated ΔGBA neurons. GAPDH was used as a loading control. Values are expressed as fold-change compared to Corr (N = 3 independent experiments **d** Quantification of average minimum duration of M-L contacts in Corr, ΔGBA, and S181-treated ΔGBA neurons. (Corr (n = 26 contacts from 6 neurons), ΔGBA (n = 26 contacts from 6 neurons), ΔGBA + S181 (n = 25 contacts from 6 neurons, N = 3 independent experiments)) (*p= 0.0178 (left); *p=0.0400 (right)). **c,d,f** One-way ANOVA followed by Tukey's multiple comparisons test.

For all quantifications, data are the means ± S.E.M. *p≤ 0.05, **p≤0.01, ***p≤0.001, ns: not significant.

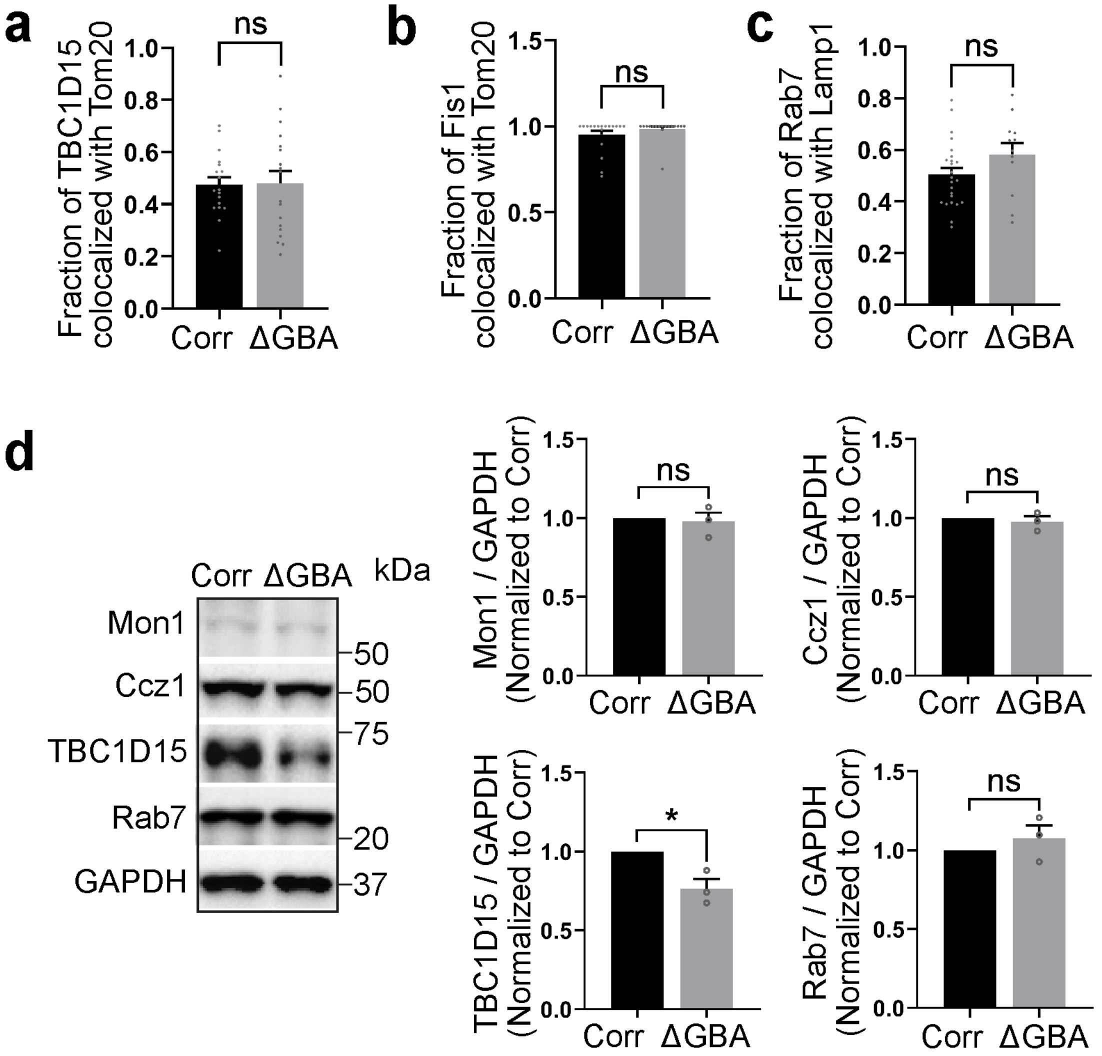

Supplementary Fig.7 Analysis of regulators of mitochondria-lysosome contact sites in *GBA1*-PD patient dopaminergic neurons.

a-c Mander's overlap coefficient (MOC) values were measured to quantify the fraction of **a** TBC1D15 colocalized with mitochondrial marker Tom20 (18 neurons per condition from N = 3 independent experiments), **b** Fis1 colocalized with mitochondrial marker Tom20 (18 Corr neurons and 17 ΔGBA neurons from N = 3 independent experiments) and **c** Rab7 colocalized with lysosomal marker Lamp1 (24 Corr neurons and 12 ΔGBA neurons from N = 3 independent experiments) from immunostained confocal images in PD patient-derived mutant *GBA1* dopaminergic neurons (ΔGBA) and its CRISPR-corrected isogenic control (Corr) neurons. **a-c** Unpaired two-sided Student's t-test. **d** Western blot analysis of Mon1 and Ccz1 (Rab7-GEF), TBC1D15 (Rab7-GAP) and Rab7 protein levels in ΔGBA and Corr neurons. Protein levels were normalized to loading control GAPDH. Values are expressed as fold-change compared to Ctrl (N = 3 independent experiments). Paired two-sided Student's t-test; *p=0.0181.

For all quantifications, data are the means ± S.E.M. *p≤ 0.05, ns: not significant.

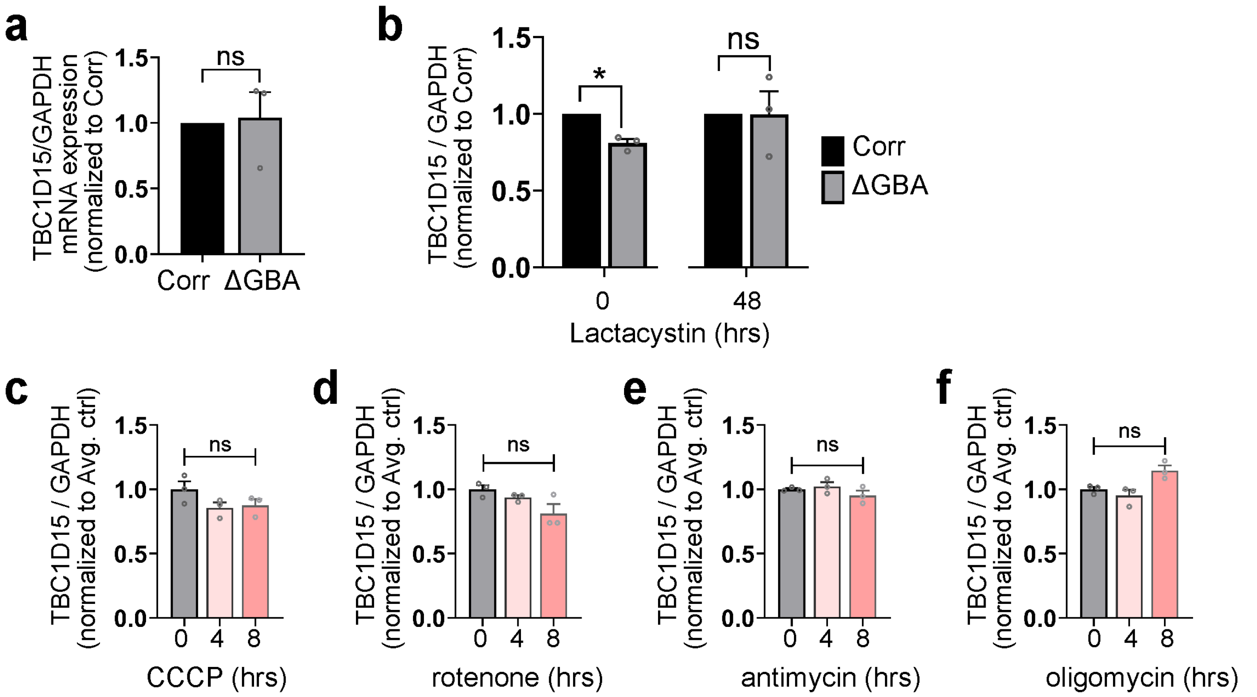

Supplementary Fig.8 Decreased TBC1D15 protein levels in *GBA1*-PD patient dopaminergic neurons.

a Quantification of TBC1D15 mRNA expression levels by RT-PCR analysis in PD patient-derived mutant *GBA1* dopaminergic neurons (ΔGBA) and its CRISPR-corrected isogenic control (Corr) neurons, showing no significant difference between Corr and ΔGBA neurons (N = 3 independent experiments). **b** Western blot analysis of TBC1D15 level in Corr and ΔGBA neurons before and after the treatment of proteasome inhibitor lactacystin (1.5μM, 48hrs) (N = 3 independent experiments), showing that inhibition of proteasomal degradation leads to similar TBC1D15 levels in ΔGBA neurons and Corr neurons. *p=0.0192 **a-b** Paired two-sided Student's t-test. **c-f** Western blot analysis of TBC1D15 level in HeLa cells treated with mitochondrial toxins, normalized to ctrl (0h): **c** CCCP (10uM); **d** rotenone (100nM); **e** Antimycin A (1uM); **f** Oligomycin (1uM) for 0, 4, 8 hrs, showing that mitochondrial dysfunction does not alter TBC1D15 protein levels. (N = 3 independent experiments) **c-f** One-way ANOVA followed by Tukey's multiple comparisons test.

For all quantifications, data are the means ± S.E.M. *p≤0.05, ns: not significant.

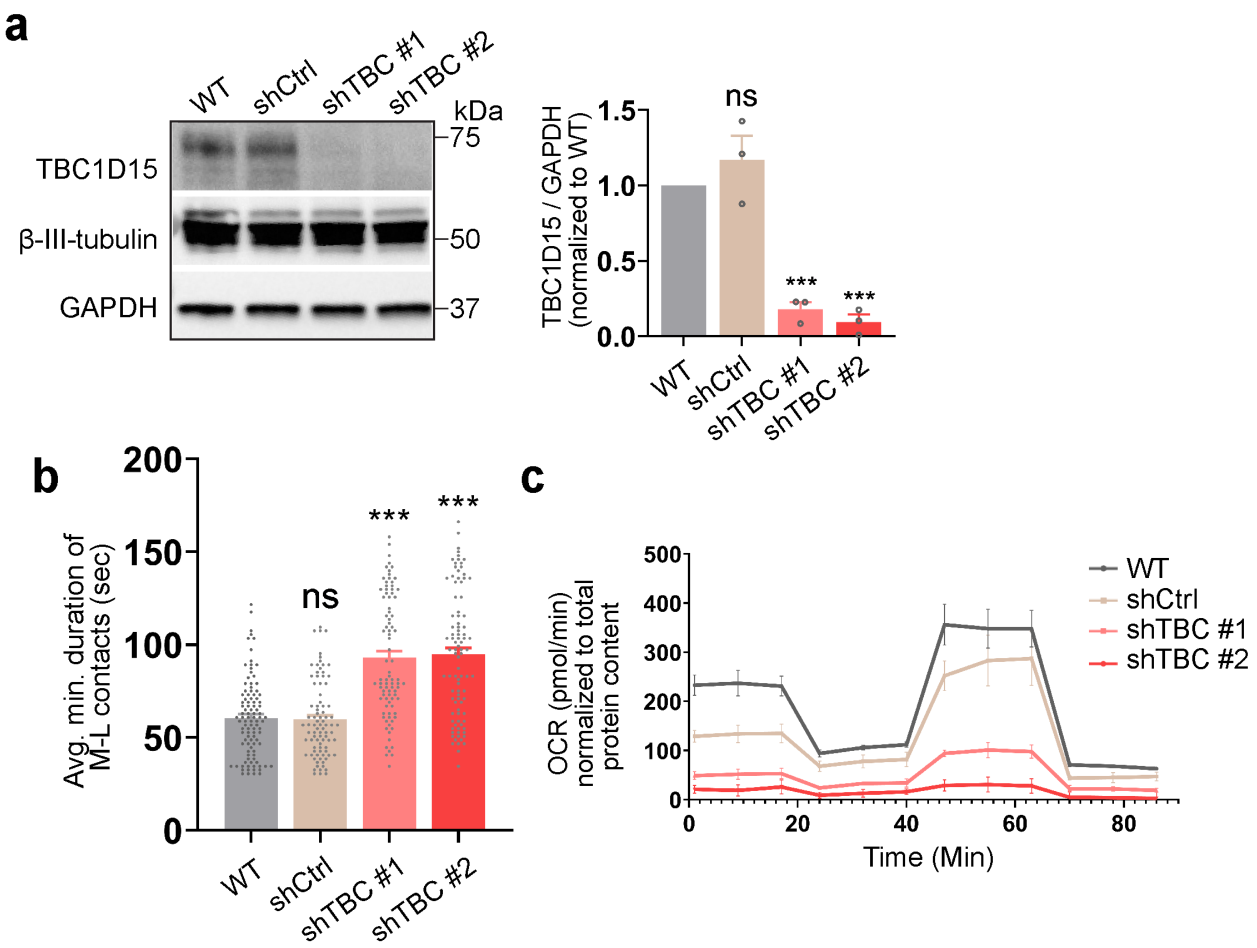

Supplementary Fig.9 Knockdown of TBC1D15 in human dopaminergic neurons disrupts mitochondria-lysosome contact dynamics and mitochondrial function.

a Neurons were infected with lentivirus expressing either non-targeting shRNA (shCtrl) or shRNAs to knock down TBC1D15 (shTBC #1 and #2) (MOI=5, 10 days). Western blot analysis shows knockdown efficiency. β-iii-tubulin and GAPDH were used as loading controls. (N = 3 independent experiments). (shTBC#1 ***p=0.0004; shTBC#2 ***p=0.0002;) **b** Quantification of average minimum duration of M-L contacts in wild-type neurons (WT, n = 101 contacts from 23 neurons), non-targeting shRNA treated neurons (shCtrl, n = 86 contacts from 21 neurons), and TBC-KD neurons (shTBC #1, n = 88 contacts from 20 neurons and shTBC #2, n = 93 contacts from 23 neurons), (N = 3 independent experiments). (shTBC#1 ***p<0.0001; shTBC#2 ***p<0.0001;) **a-b** One-way ANOVA followed by Tukey's multiple comparisons test. **c** Oxygen consumption rate (OCR) was measured by Seahorse assay and normalized to total protein content. (9 samples of each condition, N = 3 independent experiments).

For all quantifications, data are the means ± S.E.M. ***p≤0.001, ns: not significant.

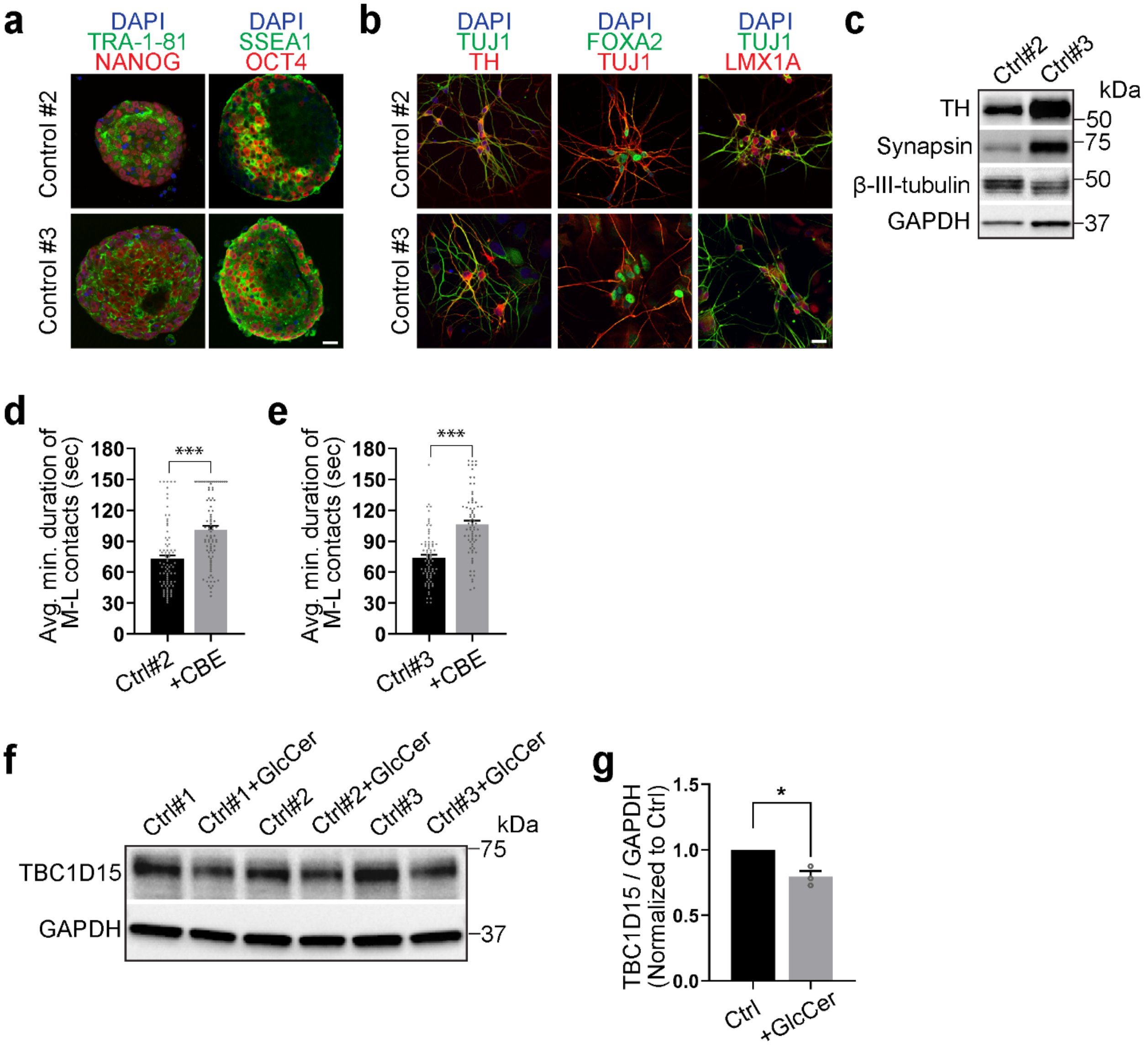

Supplementary Fig.10 Inhibition of GCase activity leading to increased GlcCer disrupts mitochondria-lysosome contact dynamics and machinery.

a Immunocytochemistry demonstrated that two additional wild-type control iPSC lines (Control #2 and Control #3) expressed pluripotency markers including Oct4, SSEA1, TRA-1-81 and Nanog. Left panel, merged images of Tra 1-81 (green), Nanog (red), and DAPI (blue) nuclear staining. Right panel, merged images of Oct4 (red), SSEA1 (green), and DAPI (blue) nuclear staining from both control lines. Scale bar, 20µm. (N = 3 independent experiments) **b-c** Characterization of wild-type control iPSC-derived dopaminergic neurons (Control #2 and Control #3). **b** Neurons were analyzed by immunofluorescence for the expression of dopamine (TH; tyrosine hydroxylase), midbrain (FOXA2, LMX1A) and neuronal cytoskeletal (Tuj1) markers at day 30. Scale bar = 20µm. (N = 3 independent experiments) **c** Western blot analysis of human dopaminergic neurons at day 50. The expression of dopaminergic neuronal markers (TH, Synapsin and β−iii Tubulin) were confirmed in two control lines. (N = 2 independent experiments) **d** CBE treatment in control neurons increased the average minimum duration of stable M-L contacts Ctrl #2

(n = 84 contacts from Ctrl #2 and n=89 contacts from +CBE neurons), (N = 3 independent experiments). **e** Ctrl #3 (n = 77 contacts from Ctrl #3 and n=75 contacts from +CBE neurons), (N = 3 independent experiments). **d-e** Unpaired two-sided Student's t-test; ***p<0.0001 **f-g** Western blot analysis of TBC1D15 in Ctrl (#1, #2, and #3) and GlcCer-treated neurons. Protein levels were normalized to loading control GAPDH. Values are expressed as fold-change compared to Ctrl (N = 3 independent experiments). Paired two-sided Student's t-test; *p=0.0401.

For all quantifications, data are the means ± S.E.M. *p≤0.05, ***p≤0.001.

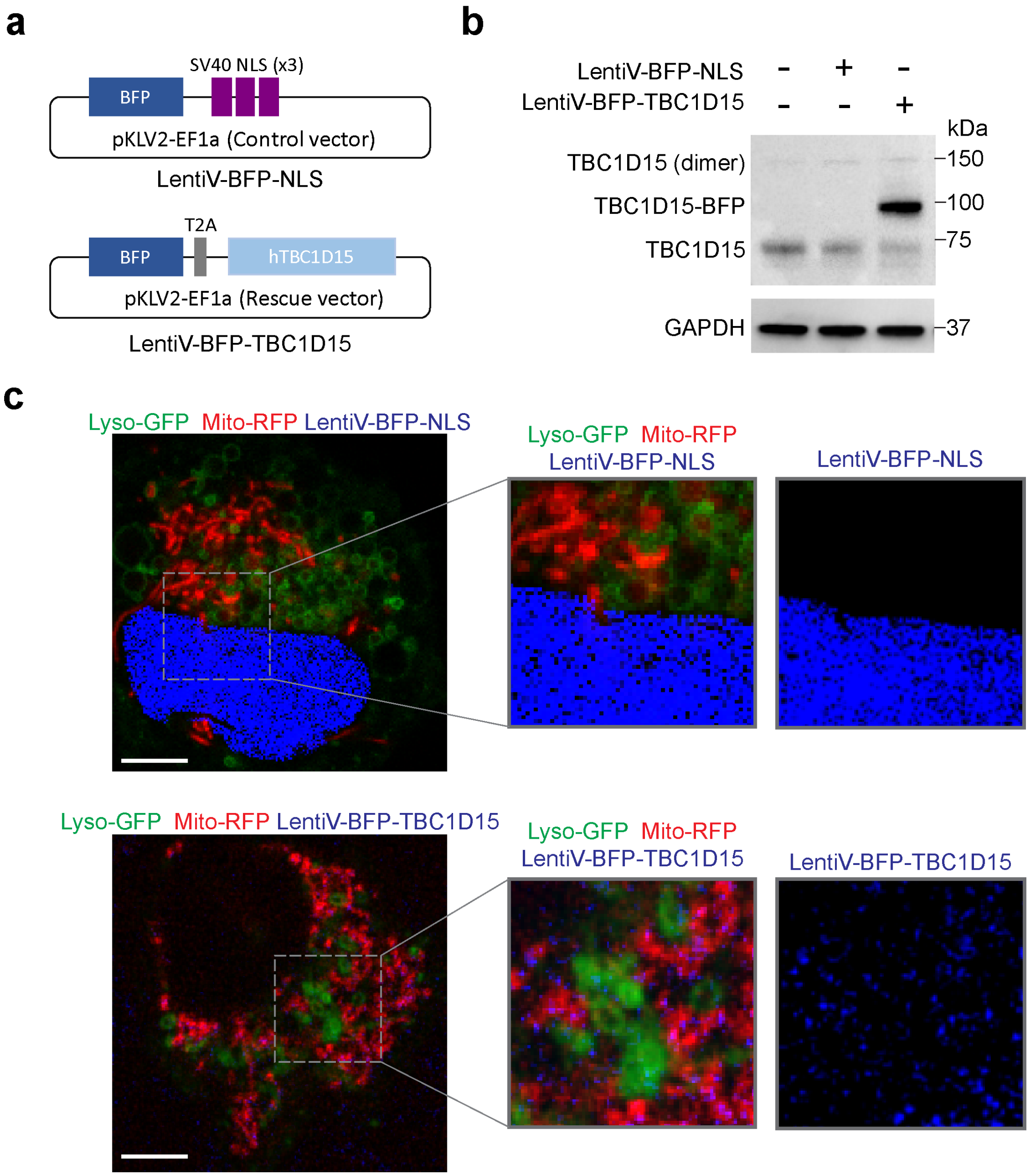

Supplementary Fig.11 Lentiviral expression of human TBC1D15 in iPSC-derived dopaminergic neurons.

a Lentiviral vector constructs expressing the BFP-NLS (vehicle control vector) or BFP-TBC1D15 (rescue vector) under control of the EF-1α promoter. **b** Western blot analysis of TBC1D15 in wild-type human iPSC-derived dopaminergic neurons transduced by lentivirus expressing nuclear-localized BFP or BFP-tagged TBC1D15. GAPDH was used as a loading control. (N = 2 independent experiments) **c** Representative confocal images of lentiviral BFP, mitochondria (Mito-RFP, red) and lysosomes (Lyso-GFP, green) in live wild-type neurons. Scale bar, 5µm. (N = 3 independent experiments).

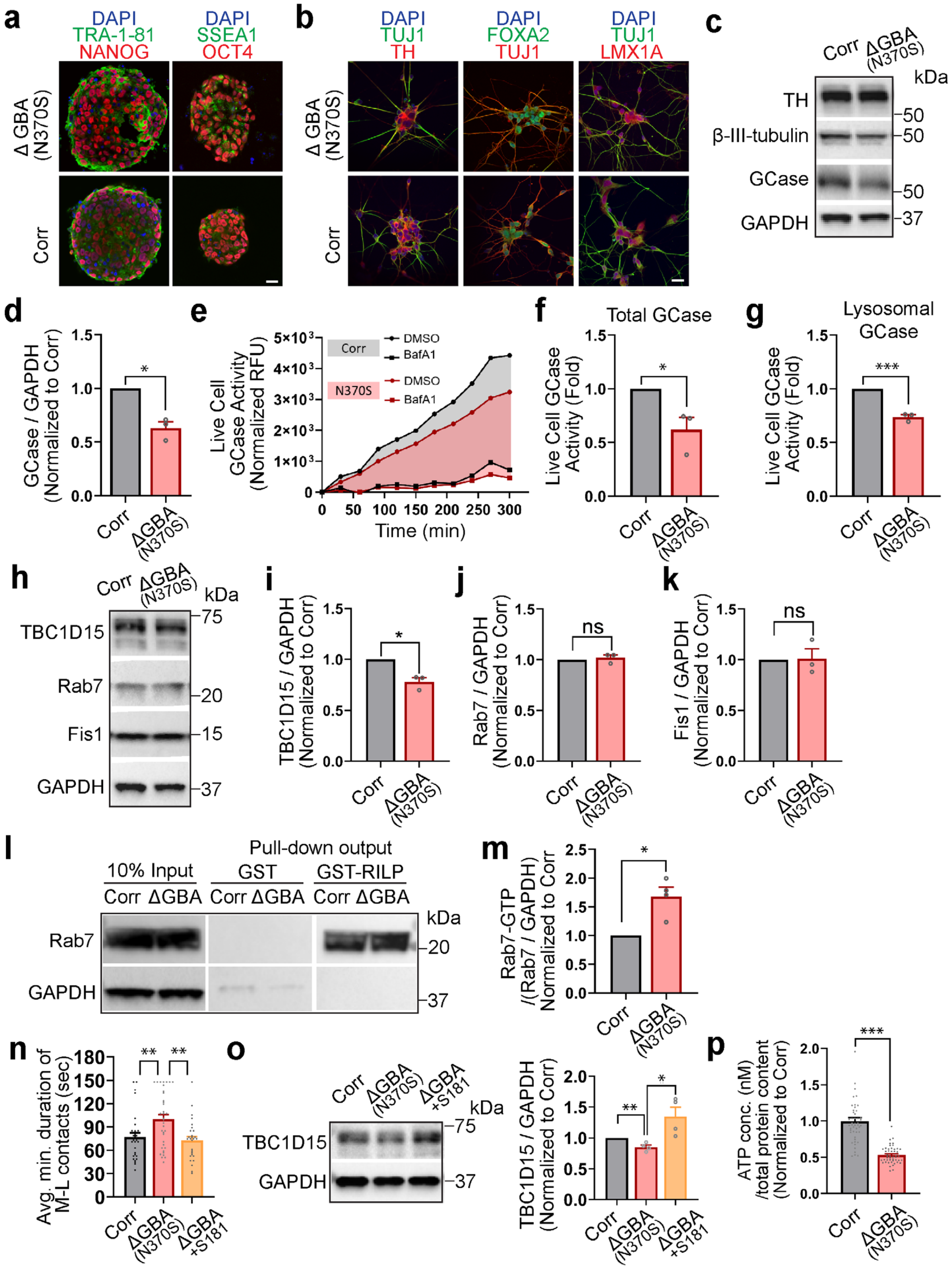

Supplementary Fig.12 Defective mitochondria-lysosome contact dynamics in mutant *GBA1* (N370S) Parkinson's patient-derived neurons.

a Immunocytochemistry demonstrated that mutant GBA1 (ΔGBA (het N370S)) and CRISPR-corrected isogenic control (Corr) induced Pluripotent Stem Cells (iPSCs) expressed pluripotency markers including Oct4, SSEA1, TRA-1-81 and Nanog. Left panel, merged images of Tra 1-81 (green), Nanog (red), and DAPI (blue) nuclear staining. Right panel, merged images of Oct4 (red), SSEA1 (green), and DAPI (blue) nuclear staining from each line. Scale bar, 20µm. (N = 3 independent experiments) **b-d** Characterization of ΔGBA

(N370S) and Corr iPSC-derived dopaminergic neurons. **b** Neurons were analyzed by immunofluorescence for the expression of dopamine (TH; tyrosine hydroxylase), midbrain (FOXA2, LMX1A) and neuronal cytoskeletal (Tuj1) markers at day 30. Scale bar = 20μm. **c** Western blot analysis of human dopaminergic neurons at day 50. The expression of dopaminergic neuronal markers (TH and β−iii Tubulin) were confirmed in Corr and ΔGBA (N370S) lines. **d** GCase levels were significantly reduced in ΔGBA (N370S) neurons (N = 3 independent experiments). Paired two-sided Student's t-test; *p=0.0249. **e-g** ΔGBA (N370S) and Corr neurons were treated with either DMSO or BafA1 and subjected to live cell GCase activity analysis. **f** Quantification of the area under each curve (AUC) demonstrates decreased total GCase activity in ΔGBA (N370S) neurons. **g** Lysosomal GCase activity was calculated by subtracting BafA1 values from DMSO. Values are expressed as fold-change compared to Corr (N = 3 independent experiments). **f-g** Paired two-sided Student's t-test; *p=0.0304; ***p=0.0002. **h-k** Western blot analysis of **i** TBC1D15, **j** Rab7 and **k** Fis1 levels in Corr and ΔGBA (N370S) neurons. Protein levels were normalized to loading control GAPDH. Values are expressed as fold-change compared to Corr (N = 3 independent experiments). Paired two-sided Student's t-test; *p=0.0344. **l-m** GST-RILP pull-down to measure GTP-bound Rab7 levels in ΔGBA (N370S) and Corr neurons. **m** Rab7-GTP levels were normalized to total Rab7 normalized to GAPDH. Values are expressed as fold-change compared to Corr (N = 4 independent experiments). Paired two-sided Student's t-test; *p=0.0242 **n** Quantification of average minimum duration of stable M-L contacts showing increased duration of stable M-L contacts in ΔGBA (N370S) neurons, which was rescued by GCase modulator S181. (n = 95 contacts from 19 neurons). One-way ANOVA followed by Tukey's multiple comparisons test; **p=0.0099(left); **p=0.0023(right). **o** Western blot analysis of TBC1D15 in Corr, ΔGBA (N370S) and S181-treated ΔGBA (N370S) neurons. GAPDH was used as a loading control. Values are expressed as fold-change compared to Ctrl (N = 4 independent experiments). One-way ANOVA followed by Tukey's multiple comparisons test; **p=0.0040(left); *p=0.0183(right). **p** Total cellular ATP content was measured and normalized to total protein content (ng/μl). Corr (n = 48 samples), ΔGBA (N370S) (n = 48 samples). (N = 3 independent experiments). Unpaired two-sided Student's t-test; ***p<0.0001.

For all quantifications, data are the means ± S.E.M. *p≤ 0.05, **p≤0.01, ***p≤0.001, ns: not significant.
