# Supplementary material for: Dysregulation of mitochondria-lysosome contacts by GBA1 dysfunction in dopaminergic neuronal models of Parkinson’s disease
Source: Nat Commun. 2021 Mar 22;12:1807. doi: 10.1038/s41467-021-22113-3 (PMC7985376; doi:10.1038/s41467-021-22113-3)
Supplement: Supplementary file 2 — Description of Additional Supplementary Files [file 41467_2021_22113_MOESM2_ESM.pdf]

Description of Additional Supplementary Files

File Names: Supplemental Movies 1-3.

Description: Live imaging of mitochondria-lysosome contacts in human neurons. Representative time-lapse confocal images of contacts between mitochondria (red; Mito-RFP) and lysosomes (green; Lyso-GFP) in wild-type live human dopaminergic neurons. Time-lapse recordings were taken at 2 sec intervals for 3-5min and played back at 5 frames/sec (10x speed). Arrows mark the site of mitochondria-lysosome contacts. Movie 1 corresponds to Fig. 1c (left); Movie 2 corresponds to Fig. 1c (middle) and shows an example of a lysosome marking the site of mitochondrial division in human neurons.

File Names: Supplemental Movies 4-6.

Description: Live imaging of mitochondria-lysosome contacts in specific compartment of human neurons. Representative time-lapse confocal images of contacts between mitochondria (red; Mito-RFP) and lysosomes (green; Lyso-GFP) in the soma (movie 4), dendrite (movie 5), and axon (movie 6) of wild-type live human dopaminergic neurons. Time-lapse recordings were taken at 2 sec intervals for 3-5min and played back at 5 frames/sec (10x speed). Arrows mark the site of mitochondria-lysosome contacts. Movies 4-6 corresponds to Fig. 2a (3 panels) in the same order.

File Names: Supplemental Movies 7-8.

Description: Live imaging of mitochondria-lysosome contacts in *GBA1*-PD patient and CRISPR-corrected isogenic control neurons. Representative time-lapse confocal images of contacts between mitochondria (red; Mito-RFP) and lysosomes (green; Lyso-GFP) in *GBA1*-PD patient (movie 8) and CRISPR-corrected isogenic control neurons (movie 7). Time-lapse recordings were taken at 2 sec intervals for 3-5min and played back at 5 frames/sec (10x speed). Arrows mark the site of mitochondria-lysosome contacts. Movies 7-8 corresponds to Fig. 3g (2 panels) in the same order.
