# Supplementary material for: Dysregulation of mitochondria-lysosome contacts by GBA1 dysfunction in dopaminergic neuronal models of Parkinson’s disease
Source: Nat Commun. 2021 Mar 22;12:1807. doi: 10.1038/s41467-021-22113-3 (PMC7985376; doi:10.1038/s41467-021-22113-3)
Supplement: Supplementary file 11 — Reporting Summary [file 41467_2021_22113_MOESM11_ESM.pdf]

nature research

Corresponding author(s): Dimitri Krainc

Last updated by author(s): Jan 27, 2021

Reporting Summary

Nature Research wishes to improve the reproducibility of the work that we publish. This form provides structure for consistency and transparency in reporting. For further information on Nature Research policies, see our Editorial Policies and the Editorial Policy Checklist.

Statistics

For all statistical analyses, confirm that the following items are present in the figure legend, table legend, main text, or Methods section.

n/a	Confirmed	
☐	☒	The exact sample size (n) for each experimental group/condition, given as a discrete number and unit of measurement
☐	☒	A statement on whether measurements were taken from distinct samples or whether the same sample was measured repeatedly
☐	☒	The statistical test(s) used AND whether they are one- or two-sided<br>*Only common tests should be described solely by name; describe more complex techniques in the Methods section.*
☐	☒	A description of all covariates tested
☐	☒	A description of any assumptions or corrections, such as tests of normality and adjustment for multiple comparisons
☐	☒	A full description of the statistical parameters including central tendency (e.g. means) or other basic estimates (e.g. regression coefficient) AND variation (e.g. standard deviation) or associated estimates of uncertainty (e.g. confidence intervals)
☐	☒	For null hypothesis testing, the test statistic (e.g. F, t, r) with confidence intervals, effect sizes, degrees of freedom and P value noted<br>*Give P values as exact values whenever suitable.*
☒	☐	For Bayesian analysis, information on the choice of priors and Markov chain Monte Carlo settings
☒	☐	For hierarchical and complex designs, identification of the appropriate level for tests and full reporting of outcomes
☒	☐	Estimates of effect sizes (e.g. Cohen's d, Pearson's r), indicating how they were calculated

Our web collection on statistics for biologists contains articles on many of the points above.

Software and code

Policy information about availability of computer code

Data collection	Nikon NIS-Elements (Nikon, AR 5.20.00) was used for Live cell imaging and Leica Application Suite X (Leica, Ver 1.8.1) was used for capturing Immunocytochemistry images.
Data analysis	Images and movies were analyzed and assembled using NIS-Elements (Nikon, Ver. 4.2 and Ver. 5.21.00) and ImageJ (NIH, Ver. 1.52p). ImageJ was used for densitometric quantification of western blots. Statistics and graphing were performed using Prism 7 (GraphPad) software. Figures were assembled in Adobe Illustrator 2019.

For manuscripts utilizing custom algorithms or software that are central to the research but not yet described in published literature, software must be made available to editors and reviewers. We strongly encourage code deposition in a community repository (e.g. GitHub). See the Nature Research guidelines for submitting code & software for further information.

Data

Policy information about availability of data

All manuscripts must include a data availability statement. This statement should provide the following information, where applicable:

- Accession codes, unique identifiers, or web links for publicly available datasets
- A list of figures that have associated raw data
- A description of any restrictions on data availability

All data that support the findings of this study are included in manuscript or are available from the authors upon reasonable request.

Field-specific reporting

Please select the one below that is the best fit for your research. If you are not sure, read the appropriate sections before making your selection.

- [x] Life sciences
- [ ] Behavioural & social sciences
- [ ] Ecological, evolutionary & environmental sciences

For a reference copy of the document with all sections, see nature.com/documents/nr-reporting-summary-flat.pdf

Life sciences study design

All studies must disclose on these points even when the disclosure is negative.

Sample size	All sample sizes are listed in detail in the figure legends and main text. No statistical method was used to determine sample sizes. Based on previous publications, a reasonable sample size was chosen to achieve statistical significance and minimize cost.
Data exclusions	In live-cell imaging experiments, any cells were excluded if they were unresolved by fluorescence signal. No data was excluded in the analysis stage.
Replication	For all statistical tests, data were from N ≥ 3 independent experiments (biological replicates). All attempts at replication were successful in all experiments. Also, additional steps were taken to ensure rigor and reproducibility, as follows: 1) scientific questions were addressed using complementary technical approaches to ensure that the findings were robust; 2) for studies involving multiple different experimental conditions in the same line, studies were performed on neurons originating from the same batch.
Randomization	Neurons from each line or condition were randomly allocated in different wells and randomly assigned into different experimental groups. Since patients' neurons were grouped based on the genotype and compared with the respective isogenic control, no additional randomization was applicable for our experiments.
Blinding	Blinding was not applicable for the characterization of mitochondria-lysosome contact sites in wild-type control neurons because all neurons were cultured in the same condition, and analyzed using the same methods. The experimenters who performed the quantification of the live cell images or immunostainings were blinded to the genotype/treatment of the samples to exclude bias.

Reporting for specific materials, systems and methods

We require information from authors about some types of materials, experimental systems and methods used in many studies. Here, indicate whether each material, system or method listed is relevant to your study. If you are not sure if a list item applies to your research, read the appropriate section before selecting a response.

Materials & experimental systems

n/a	Involved in the study
☐	☒ Antibodies
☐	☒ Eukaryotic cell lines
☒	☐ Palaeontology and archaeology
☒	☐ Animals and other organisms
☒	☐ Human research participants
☒	☐ Clinical data
☒	☐ Dual use research of concern

Methods

n/a	Involved in the study
☒	☐ ChIP-seq
☒	☐ Flow cytometry
☒	☐ MRI-based neuroimaging

Antibodies

Antibodies used	All antibody dilutions are detailed in the Methods section. For western blot analysis, rabbit anti-Rab7 (Cell Signaling, #D95F2; Abcam, #ab137029); rabbit anti-TBC1D15 (Sigma Aldrich, #SAB2701508); rabbit anti-Fis1 (Alexis, # ALX-210-1037-0100); mouse anti-GAPDH (Millipore, clone 6C5, #MAB374); mouse anti-GBA (Abnova, clone 2E2, #H00002629-M01); rabbit anti-TH (Calbiochem, #657012); mouse anti-Synapsin (Santa Cruz, #sc-398849); mouse anti-βIII tubulin (tuj1) (Biolegend, #801202); rabbit anti-AMPKα (Cell Signaling, #2532); rabbit anti-Phospho-AMPKα (Cell Signaling, #2535); mouse anti-Ccz1 (Santa Cruz, #sc-514290); goat anti-Mon1 (Abcam, #ab103919); mouse anti-Tom20 (Abcam, #ab56783); mouse anti-Lamp1 (Santa Cruz, #sc-20011) and the secondary antibodies goat anti-mouse and goat anti-rabbit (Jackson ImmunoResearch lab, #115-035-146, #111-035-144) were used.<br><br>For immunocytochemistry, rabbit anti-Oct4 (Abcam, #19857); mouse anti-SSEA1 (Millipore, MAB#4304); goat anti-Nanog (R&D systems, #AF1997); rabbit anti-Tra-1-81 (Millipore, #MAB4381); rabbit anti-TH (Calbiochem, #657012); rabbit anti-β-III-tubulin (Biolegend, #802001); mouse anti-β-III-tubulin (Biolegend, #801202); rabbit anti-Lmx1a (Milipore, MAB#10533); mouse anti-FoxA2 (Santa Cruz, #sc-101060); rabbit anti-Tom20 (Abcam, #78547); mouse anti-Lamp1 (Santa Cruz, #sc-20011); chicken anti-Map2 (Novus Biological, #NB300213); rabbit anti-Tau (DAKO, #A002401-2); rabbit anti-GlcCer (Glycobiotech, #RAS_0011) and Alexa fluorophore-conjugated secondary antibodies from Molecular Probes (Invitrogen), Donkey anti-rabbit 488 (#A21206), Donkey anti-mouse 488 (#A21202), Goat anti-mouse 488 (#A11029), Goat anti-rabbit 568 (#A11011), Donkey anti-rabbit 568 (#A10042), Donkey anti-mouse 568(#A10037), Goat anti-chicken 647 (#A21449) were used.

For Proximity Ligation Assay (PLA), rabbit anti-Tom20 (Abcam, #78547) and mouse anti-Lamp1 (Santa Cruz, #sc-20011) were used.

Validation

We chose antibodies that have been highly cited and validated by multiple labs. In addition to data provided by manufacturers shown in the following, antibodies were routinely validated for use in the system under study.

According to the manufacturer's website:
Rab7 (Cell Signaling, #D95F2) reacts with human samples and has been cited in 213 publications.
Rab7 (Abcam, #ab137029) reacts with human samples and has been cited in 44 publications. Knockout validation was done by the manufacturer.
TBC1D15 (Sigma Aldrich, #SAB2701508) reacts with human samples.
Fis1 (Alexis, # ALX-210-1037-0100) reacts with human samples and has been cited in 6 publications.
GAPDH (Millipore, #MAB374) reacts with human samples and has been cited in 2608 publications.
GBA (Abnova, #H00002629-M01) reacts with human samples and has been cited in 6 publications.
TH (Calbiochem, #657012) reacts with human samples and has been cited in 6 publications and cited in several papers from our lab including Burbulla et al (2017).
Synapsin (Santa Cruz, #sc-398849) reacts with human samples and has been cited in 4 publications.
βIII tubulin (tuj1) (Biolegend, #801202) reacts with human samples and has been cited in 454 publications.
AMPKα (Cell Signaling, #2532) reacts with human samples and has been cited in 1189 publications.
Phospho-AMPKα (Cell Signaling, #2535) reacts with human samples and has been cited in 1644 publications.
Ccz1 (Santa Cruz, #sc-514290) reacts with human samples and has been cited in 3 publications.
Mon1 (Abcam, #ab103919) reacts with human and validated in Jurkat cell lysate by the manufacturer.
Tom20 (Abcam, #ab56783) reacts with human samples and has been cited in 98 publications.
Lamp1 (Santa Cruz, #sc-20011) reacts with human samples and has been cited in 305 publications.
Tom20 (Abcam, #78547) reacts with human samples and has been cited in 45 publications.
Map2 (Novus Biological, #NB300213) reacts with human samples and has been cited in 59 publications.
Tau (DAKO, #A002401-2) reacts with human samples and has been cited in 172 publications.
GlcCer (Glycobiotech, #RAS_0011) reacts with human samples and validated in Mazzulli et al (2011).

All iPSC markers Oct4 (Abcam, #19857), SSEA1 (Millipore, MAB#4304), Nanog (R&D systems, #AF1997), and Tra-1-81 (Millipore, #MAB4381) and dopaminergic neuron markers TH (Calbiochem, #657012), β-III-tubulin (Biolegend, #802001 and #801202)), Lmx1a (Milipore, MAB#10533), FoxA2 (Santa Cruz, #sc-101060) have been validated by our lab and cited in several papers including Burbulla et al (2017), Nguyen et al (2018) and Ysselstein et al (2019).

Eukaryotic cell lines

Policy information about cell lines

Cell line source(s)

All primary patient fibroblasts were obtained from the Northwestern University Biorepository and were reprogrammed into iPSCs through the Northwestern University Stem Cell Core Facility. Isogenic control iPSC lines were generated by correcting the mutation using CRISPR/Cas9 protocols.
HEK 293FT cells were purchased from Invitrogen (#R70007) and HeLa cells were purchased from ATCC.

Authentication

All iPSC cell lines were subjected to karyotyping after reprogramming. All specific point mutations in iPSCs were confirmed by sequencing in our previous studies.
Hela cells were previously authenticated by cytochrome c oxidase subunit I (COI) and short tandem repeat (STR) testing.

Mycoplasma contamination

All cell lines including iPSCs, iPSC-derived neurons, HEK 293FT and HeLa cells were routinely tested for mycoplasma testing using a PCR based detection (Venor GeM Mycoplasma Detection Kit (Sigma, #MP0025)) and confirmed to be negative for mycoplasma contamination.

Commonly misidentified lines
(See ICLAC register)

None
